# Four new suomilides isolated from the cyanobacterium *Nostoc* sp. KVJ20 and proposal of their biosynthetic origin

**DOI:** 10.3389/fmicb.2023.1130018

**Published:** 2023-04-20

**Authors:** Yannik K.-H. Schneider, Anton Liaimer, Johan Isaksson, Oda S. B. Wilhelmsen, Jeanette H. Andersen, Kine Ø. Hansen, Espen H. Hansen

**Affiliations:** ^1^Marbio, Faculty of Biosciences, Fisheries and Economics, UiT—The Arctic University of Norway, Tromsø, Norway; ^2^Department of Arctic and Marine Biology, Faculty of Biosciences, Fisheries and Economics, UiT—The Arctic University of Norway, Tromsø, Norway; ^3^Department of Chemistry, Faculty of Natural Sciences, UiT—The Arctic University of Norway, Tromsø, Norway

**Keywords:** *Nostoc*, cyanobacteria, natural products, protease inhibitor, biosynthesis, secondary metabolites, aeruginosin, suomilide

## Abstract

The suomilide and the banyasides are highly modified and functionalized non-ribosomal peptides produced by cyanobacteria of the order Nostocales. These compound classes share several substructures, including a complex azabicyclononane core, which was previously assumed to be derived from the amino acid tyrosine. In our study we were able to isolate and determine the structures of four suomilides, named suomilide B – E (**1**–**4**). The compounds differ from the previously isolated suomilide A by the functionalization of the glycosyl group. Compounds **1**–**4** were assayed for anti-proliferative, anti-biofilm and anti-bacterial activities, but no significant activity was detected. The sequenced genome of the producer organism *Nostoc* sp. KVJ20 enabled us to propose a biosynthetic gene cluster for suomilides. Our findings indicated that the azabicyclononane core of the suomilides is derived from prephenate and is most likely incorporated by a proline specific non-ribosomal peptide synthetase-unit.

## 1. Introduction

Cyanobacteria are well known for being prolific producers of a broad range of bioactive secondary metabolites, some of which are unique to cyanobacteria (Nunnery et al., 2010). Some cyanobacteria have been recognized for the toxins they produce, which are capable of causing severe intoxications in humans and animals (Dittmann and Wiegand, 2006). One of the most prominent groups of such toxins is the microcystins, a group of phosphatase inhibitors, which are problematic when they enter drinking water supplies during dense cyanobacterial blooms (Namikoshi and Rinehart, 1996). The diverse cyanobacterial secondary metabolites are products of different biosynthetic machineries such as non-ribosomal peptide synthetases (NRPS) and polyketide synthases (PKS), but there are also peptides that are ribosomally synthesized and posttranslationally modified, the so called ribosomally synthetized and post-translationally modified peptides (RiPP) (Kehr et al., 2011; Li and Rebuffat, 2020). For the investigation of the biosynthesis of microbial metabolites, genome mining tools have been extensively used in the field of natural products in general and cyanobacterial natural products in particular (Micallef et al., 2015). A very powerful strategy to identify new secondary metabolites and their biosynthetic pathways is the integration of metabolomic and genomic studies combining the strengths of both techniques (Kleigrewe et al., 2015; Caesar et al., 2021).

**Figure 1:**
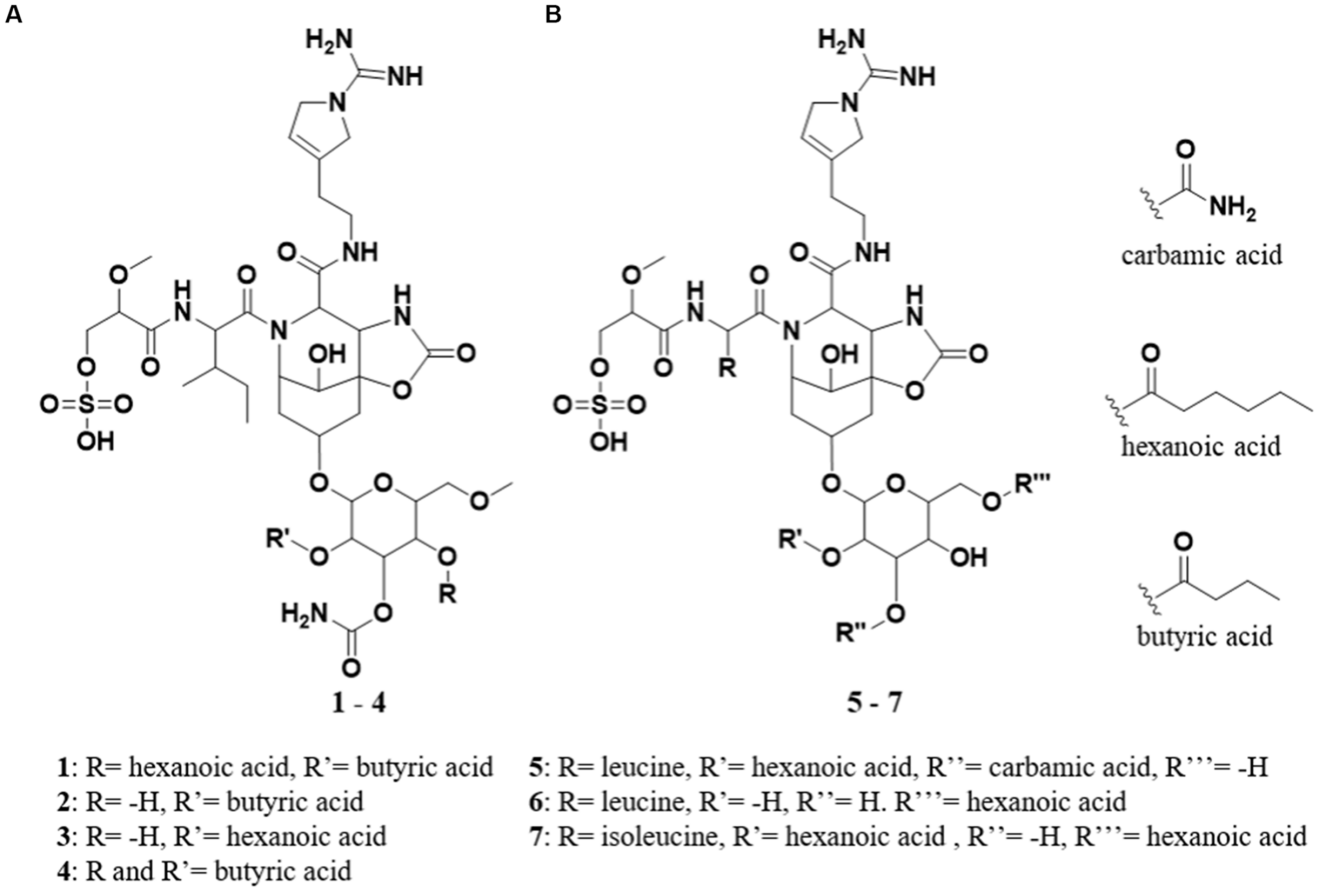
**(A)** Structures of suomilide B – E (**1**–**4**). **(B)** The previously isolated molecules banyasides A and B (**5** and **6**) and suomilide (**7**). All molecules share an Abn (azobicyclononane) core and an Aeap-moiety [1-amino-2-(N-amidino-Δ3-pyrrolinyl)ethyl], which also can be observed in the aeruginosins, as well as leucine and glycosylation. Suomilide differs from the banyasides by incorporation of isoleucine instead of leucine. The banyasides differ in the modification of their glycons (α-glucose for **4**, **5**, and **6**).

**Figure 2:**
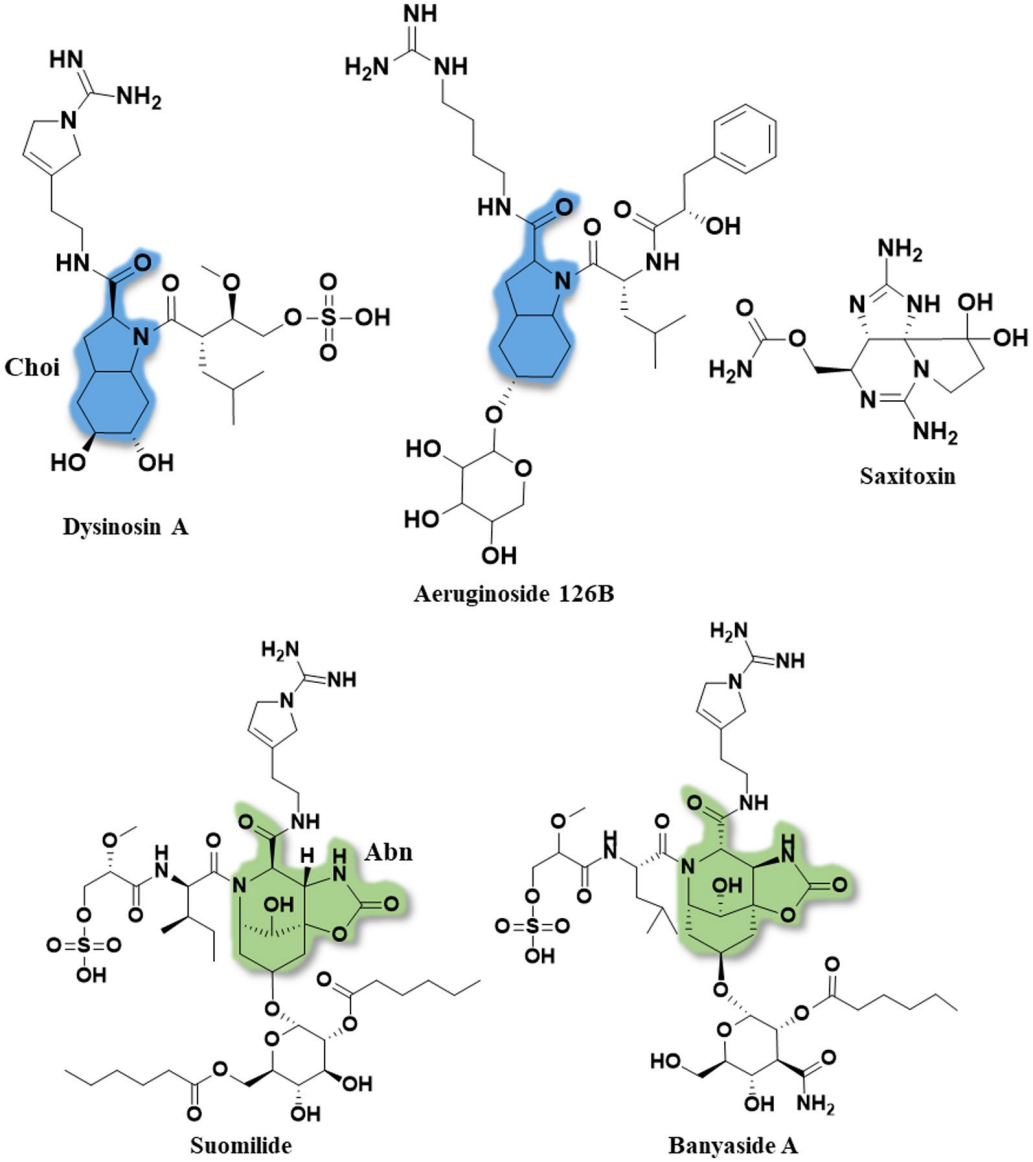
The full structures of dysinosin A, aeruginoside 126B, saxitoxin, suomilide (**7**) and banyaside A (**5**). The 2-carboxyl-6-hydroxyoctahydroindole (Choi) is marked in blue and the azabicyclononane (Abn) is highlighted in green. Note the structural similarities between the cyanobacterial metabolites, consisting of a bi-, or tri-cyclic core unit decorated with Aeap or 2-O-methylglyceric acid 3-O-sulfate (MgS).

In 1997, a new glycoside was isolated from the non-toxic cyanobacterium *Nodularia spumigena*. The structure of the compound was elucidated and named suomilide (**7**) (Figure 1A; Fujii et al., 1997), but its bioactivity was not investigated until 2021 when its potent trypsin inhibiting activity, the putative biosynthetic gene cluster (BGC) and biosynthesis were described by Ahmed et al. (2021) revealing a close similarity to aeruginoside and dysinosin BGCs, reflecting the apparent structural similarity of the compounds (see Figure 2). In 2005, two compounds with high structural similarity to suomilide, banyasides A and B (**5** and **6**) (Figure 1B), were isolated from a bloom of the cyanobacterium *Nostoc* sp. (Pluotno and Carmeli, 2005). When comparing the aglycon of **7** to the aglycon of **5** and **6**, they differ in one amino acid residue; leucine in **5** and **6** and isoleucine in **7**. The configuration of the leucine has been shown to be d in **5** and **6**, in which was also the case for **7** (Fujii et al., 1997; Pluotno and Carmeli, 2005). The difference between **5** and **6** is the modification of the glycosyl unit; **5** is esterified with hexanoic acid and carbamic acid, whereas **6** is esterified with hexanoic acid at different positions (Figure 1B).

Beside their potential for production of secondary metabolites, cyanobacteria have the capability of fixating atmospheric nitrogen. This feature is utilized by several land plants, ranging from mosses to angiosperms, which have developed the ability to attract diazotrophic *Nostoc* as their symbiotic partners (Nilsson et al., 2000). In a study from Liaimer et al. (2016) isolated a number of diverse *Nostoc* sp. strains, including KVJ20 from the symbiotic organs of the liverwort *Blasia pusilla* found at two different habitats in northern Norway. Mass spectrometric analysis of extracts from KVJ20 cultures indicated that they contained previously undescribed banyaside and suomilide like (bsl) molecules.

The crude extracts of KVJ20 showed anti-proliferative activity against a human melanoma cell line (A2058) and a human lung fibroblast cell line (MRC5) (Liaimer et al., 2016). These observations prompted a chemical investigation on the extract, which in turn led to the isolation of four bsl compounds. The draft genome of KVJ20 was published in 2019 (Halsør et al., 2019), and this enabled us to combine chemical analysis of the culture extracts with genome mining for biosynthetic gene clusters. In this study, we present the chemical and biological characterization of four novel suomilide-like compounds, suomilide B – E (**1**–**4**, Figure 1A). In addition, we investigated the *bsl* gene cluster coding for biosynthetic enzymes involved in the suomilide biosynthesis.

## 2. Materials and methods

### 2.1. General experimental procedures

UPLC-ESI-HR-MS/MS-analysis of the samples was done using an Acquity I-class UPLC (Waters, Milford, MA, United States) coupled to a PDA detector and Vion^®^ IMS QToF (Waters), UPLC-ESI-IMS-MS analysis was done using the same Vion^®^ IMS QToF in IMS (ion mobility spectrometry)-mode. For the purification of compounds via preparative HPLC a 600 HPLC pump, a 3,100 mass spectrometer, a 2,996 photo diode array detector and a 2,767 sample manager were used with as election of columns (all Waters). NMR spectra were recorded using a Bruker Avance III HD spectrometer (Bruker, Billerica, MA, United States) operating at 599.90 MHz for ^1^H and 150.86 MHz for ^13^C. For the readout of bioassays in 96-well plates a 1,420 Multilabel Counter VICTOR3^™^ (Perkin Elmer, Waltham, MA, United States) was used.

### 2.2. Origin of isolate and genome sequencing

The isolate was collected at 69,64° N 18,73°E, Kvaløya island, Northern Norway and has been termed KVJ20 (Liaimer et al., 2016), its draft genome was sequenced and published in 2019 (Halsør et al., 2019).

### 2.3. Cultivation and extraction of *Nostoc* sp. KVJ20

The subject of this investigation, the cyanobacteria *Nostoc* sp. KVJ20, was maintained as described previously (Nilsson et al., 2000; Mihali et al., 2009). The scale-up cultures were grown for 5 weeks in 1 L bottles with constant aeration. The cultures were illuminated with 30 μmol/m^2^/s using a 36 W/77 Osram Fluora light source. Following cultivation, the cells were harvested by centrifugation, the pellet was freeze dried and sonicated in 100% methanol (MeOH), centrifuged again and the methanol supernatant was collected. The pellet was re-extracted with 50% MeOH *aq*. and 100% ddH_2_O, without additional sonification. The extracts were pooled and reduced to dryness at 40°C *in vacuo*.

### 2.4. Compound identification and dereplication

UPLC-HR-MS/MS data for dereplication and structure elucidation was recorded. The chromatographic separation was performed using an Acquity C18 UPLC column (1.7 μm, 2.1 mm × 100 mm) (Waters). Mobile phases consisted out of ddH_2_O produced by the in-house Milli-Q system for mobile phase A and acetonitrile (HiPerSolv, VWR) as mobile phase B, both containing 0.1% formic acid (v/v) (33,015, Sigma). The gradient was run from 10 to 90% B over 12 min at a flow rate of 0.45 mL/min. Samples were run in ESI+ and ESI- ionization modes. The data was processed and analyzed using UNIFI 1.9.4 (Waters). Calculation of exact ion masses was done by using ChemCalc (Patiny and Borel, 2013).

### 2.5. Isolation of compounds 1–4

#### 2.5.1. Isolation protocol

Isolation of the molecules from the extract was done using a semi preparative HPLC system. The columns used for isolation were Sunfire RP-18 preparative column (10 μm, 10 mm × 250 mm) and XSelect CSH preparative Fluoro-Phenyl column (5 μm, 10 mm × 250 mm), both columns were purchased from Waters. The mobile phases for the gradients were A [ddH_2_O with 0.1% (v/v) formic acid] and B [acetonitrile with 0.1% (v/v) formic acid], flow rate was set to 6.0 mL/min for both columns. Acetonitrile (Prepsolv^®^, Merk KGaA, Darmsatdt, Germany) and formic acid (33,015, Sigma) were purchased in appropriate quality, ddH_2_O was produced with the in-house Milli-Q^®^ system. For the MS-detection of the eluting compounds 1% of the flow was split and blended with 80% MeOH in ddH_2_O (v/v) acidified with 0.2% formic acid (Sigma) and directed to the ESI-quadrupole-MS. The fractions were collected by mass triggered fraction collection and the respective fractions were reduced to dryness under reduced pressure and by vacuum centrifugation, both at 40°C. The gradient for the first purification using the RP-18 column was 20 to 100%B in 10 min, retention times for the compounds were: **1**: 7.58 min; **2**: 5.65 min; **3**: 6.42 min; **4**: 6.90 min. For the second purification using the fluoro-phenyl column a gradient from 10 to 100%B in 15 min was used. Retention times of the respective compounds were: **1**: 11.36 min; **2**: 9.11 min; **3**: 10.21 min; **4**: 10.54 min.

#### 2.5.2. NMR spectroscopy

Structure Elucidation. NMR data of the compounds was recorded on a Bruker Avance III HD spectrometer equipped with an inverse detected TCI probe with cryogenic enhancement on ^1^H, ^2^H and ^13^C. Operating frequencies were 599.90 MHz for ^1^H and 150.86 MHz for ^13^C. For taking up the spectra the samples were dissolved in DMSO-d_6_ (NMR grade, Sigma) and recorded at 298 K. All experiments were recorded using standard pulse sequences for Proton, Presat, Carbon, DQF-COSY, HSQC, HMBC, H2BC, NOESY and ROESY (gradient selected and adiabatic versions, with matched sweeps where applicable) in Topspin 3.5pl7 and processed in Mnova 12.0.0. The solvent peak of DMSO-d_6_ was used to reference the spectra. HR-MS data were recorded on the same instrument detailed in the “Compound identification and dereplication” section.

### 2.6. Biological characterization of 1–4

#### 2.6.1. Antibacterial assay

To determine and quantify potential anti-microbial activity, a bacterial growth inhibition assay in liquid media was used. The samples were tested against *Staphylococcus aureus* (ATCC 25923), *Escherichia coli* (ATCC 259233), *Enterococcus faecalis* (ATCC 29122), *Pseudomonas aeruginosa* (ATCC 27853), *Streptococcus agalactiae* (ATCC 12386) and Methicillin resistant *Staphylococcus aureus* (MRSA) (ATCC 33591). *S. aureus*, MRSA, *E. coli* and *P. aeruginosa* were grown in Muller Hinton broth (275730, Becton, Dickinson and Company). *E. faecalis* and *S. agalactiae* were cultured in brain hearth infusion broth (53286, Sigma). Fresh bacteria colonies were transferred to the respective medium and incubated at 37°C overnight. The bacterial cultures were diluted to a culture density representing the log phase and 50 μL/well were pipetted into a 96-well microtiter plate (734–2097, Nunclon^™^, Thermo Scientific, Waltham, MA, United States). The final cell density was 1500–15,000 CFU/well. The compound was diluted in 2% (v/v) DMSO in ddH_2_O, the final assay concentration was 50% of the prepared sample, since 50 μL of sample in DMSO/water were added to 50 μL bacterial culture. After adding the samples to the plates, they were incubated over night at 37°C and the growth was determined by measuring the optical density at *λ* = 600 nm (OD600) with a 1420 Multilabel Counter VICTOR3^™^ (Perkin Elmer). A water sample was used as reference control, growth medium without bacteria was used as a negative control and a dilution series of gentamycin (A2712, Merck) from 32 to 0.01 μg/mL was used as positive control and visually inspected for bacterial growth. The positive control was used as system suitability test and the results of the antimicrobial assay were only considered valid when positive control was passed. The final concentration of DMSO in the assays was ≤2% (v/v) known to have no effect in the tested bacteria.

#### 2.6.2. Antibiofilm assay

For testing the inhibition of biofilm formation *Staphylococcus epidermidis* (ATCC 35984) was grown in Tryptic Soy Broth (TSB, 105459, Merck, Kenilworth, NJ, United States) overnight at 37°C. The overnight culture was diluted in fresh medium with 1% glucose (D9434, Sigma-Aldrich) (glucose was added for the induction of biofilm formation by *Staphylococcus epidermidis*) before being transferred to a 96-well microtiter plate; 50 μL/well were incubated overnight with 50 μL of the test compound dissolved in 2% (v/v) DMSO aq. added in duplicates. During the over-night culture, *S. epidermidis* was allowed to form a bacterial biofilm within the wells. The bacterial culture was removed from the plate and the plate was washed with ddH_2_O to remove remaining culture. The biofilm adhering within the wells of the 96 well plates was fixed at 65°C for 1 h before 70 μL 0.1% crystal violet (115,940, Merck Millipore) was added to the wells for 10 min of incubation to stain the biofilm. Excess crystal violet solution was then removed and the plate dried for 1 h at 65°C. Seventy microliters of 70% EtOH were then added to each well and the plate incubated on a shaker for 5–10 min to dissolve the stain carried by the biofilm. Inhibition of biofilm formation was assessed by the presence of violet color from the stained biofilm and was measured at 600 nm absorbance using a 1,420 Multilabel Counter VICTOR3 TM. Fifty microliters of a non-biofilm forming *Staphylococcus haemolyticus* (clinical isolate 8-7A, University hospital, UNN, Tromsø, Norway) mixed in 50 μL autoclaved ddH_2_O water was used as a control; 50 μL *S. epidermidis* mixed in 50 μL autoclaved ddH_2_O water was used as the control for biofilm formation; and 50 μL TSB with 50 μL autoclaved ddH_2_O water was used as a medium blank control.

#### 2.6.3. Cytotoxicity assays

The inhibitory effect of compounds was tested using MTS *in vitro* cell proliferation assays against two malignant and one non-malignant cell line. The malignant cell lines were human melanoma A2058 (ATCC, CLR-1147^™^) and acute myeloid leukemia MOLM 13 (Matsuo et al., 1997), as cell line for the general cytotoxicity assessment, non-malignant MRC5 lung fibroblast cells (ATCC CCL-171^™^) were used. The cells were cultured and assayed in Roswell Park Memorial Institute medium (RPMI-16040, FG1383, Merck) containing 10% (v/v) Fetal Bovine serum (FBS, 50115, Biochrom, Cambridge, United Kingdom). The cell-concentration was 4,000 cells/well for the lung fibroblast cells and 2,000 cells/well for the cancer cells. After seeding, the cells were incubated 24 h at 37°C and 5% CO_2_. The medium was then replaced with fresh RPMI-1640 medium supplemented with 10% (v/v) FBS and gentamycin (10 μg/mL, A2712, Merck). After adding 10 μL of sample diluted in 2% (v/v) DMSO in ddH2O the cells were incubated for 72 h at 37°C and 5% CO2. For assaying the viability of the cells 10 μL of CellTiter 96AQueous One^®^ Solution Reagent (G3581, Promega, Madison, WI, United States) containing tetrazolium [3-(4,5-dimethylthiazol-2-yl)-5-(3-carboxymethoxyphenyl)-2-(4-sulfophenyl)-2H-tetrazolium, inner salt] and phenazine ethosulfate was added to each well and incubated for 1 h. The tests were executed with three technical replicates and were repeated twice. The plates were read using a DTX 880 plate reader by measuring the absorbance at *λ* = 485 nm. The cell viability was calculated using the media control. As a negative control RPMI-1640 with 10% (v/v) FBS was used and 0.5% Triton^™^ X-100 (Sigma-Aldrich) was used as a positive control. The data was processed and visualized using GraphPad Prism 8.

### 2.7. Genome and gene-cluster analysis

The recently published genome of *Nostoc* KVJ20 (Halsør et al., 2019) was submitted to antiSMASH (Medema et al., 2011). Genes predicted to belong to the aeruginosin biosynthetic gene clusters were found at the edges of several contigs. Therefore, we have undertaken analysis of additional data acquired in connection to the previous genome study and processed in the same way (Halsør et al., 2019). We were able to find a contig containing the entire operon which was verified again by antiSMASH. The *bsl*-operon was deposited within GenBank and can be retrieved under the following accession number: MT269816.

### 2.8. RNA isolation and gene expression studies

As an addition to the genome-wide BGC survey we have conducted a gene expression study described in detail within the Supplementary Information S30. Along with *bslA* gene, we investigated expression patterns for all other 18 BGCs as well as *nifH*, *avaK* and *gvpC* indicative of diazotrophic growth, akinete and hormogonia differentiation, respectively. In addition to the standard cultivation condition, the cultures were subjected to nitrogen, phosphate or iron depletion, and to the presence of competitor strains. The comprehensive data is given in the Supplementary material.

## 3. Results and discussion

### 3.1. Compound identification and dereplication

Investigation of the methanol–water extract of KVJ20 cells using UHPLC-IMS-MS led to the identification of four compounds with a common fragment at *m/z* 610.3203 [M + H]^+^ (C_27_H_44_N_7_O_9_, calcd. *m/z* = 610.3201, mass error: 0.33 ppm, see Supplementary material S1.1). This mass and calculated elemental composition, are identical to the desulfo-aglycon moiety of **5**–**7** (see Supplementary material S1.6), which indicates that the compounds belong to the bsl family of molecules. The tentative identification of the new compounds’ structural relationship to the banyasides was supported by comparing their obtained MS spectra to the published MS spectrum of synthetic **6** (Schindler et al., 2010). Signals of a neutral loss of 80 u in ESI+ (see Supplementary material S1.1) indicated that the molecules were carrying a sulfate group. The supernatants of the bacterial cultures were analyzed for the presence of the compounds described above, but none of them were detected, indicating that they were not excreted by the cells to the growth medium. In addition to compounds **1**–**4**, dereplication of the cyanobacterial extract gave a hit in the ChemSpider database for elemental composition and one common fragment of the anabeanopeptin-like cyclic peptide schizopeptin at m/z 792.46506 [M + H]^+^ (calcd. m/z = 792.46599, C_42_H_62_N_7_O_8_), fragmentation and elemental compositions fitted schizopeptin 791 (see Supplementary materials S2, S3; Reshef and Carmeli, 2002). Schizopeptin has not been reported for this strain previously, but as schizopeptin is well described in literature, the peptide was not selected for isolation in this study.

### 3.2. Isolation and chemical characterization of the compounds 1–4

Compounds **1**–**4** were isolated using mass-guided fractionation on preparative HPLC from 29.4 g dry mass of lyophilizated cyanobacteria from 10 L of culture. For the first purification step, the compounds were separated using a C18 reversed phase column. The collected fractions were reduced to dryness at 40°C *in vacuo*. The fractions were dissolved in DMSO or methanol (**1** dissolved poorly in methanol, but well in DMSO after extensive shaking, **2**–**4** dissolved well in methanol), and isolated in a second step using a fluoro-phenyl reversed phase column. The yields were: **1**: 9.8 mg; **2**: 4.1 mg; **3**: 5.9 mg; **4**: 2.6 mg.

#### 3.2.1. Isolated compounds

Suomilide B (**1**): white powder (9.8 mg); HRESIMS *m/z* 1075.4496 [M – H]^−^ (calcd. for C_45_H_71_N_8_O_20_S, 1075.4510) Mass error: 1.30 ppm. Retention Time_UPLC_: 4.14 min; CCS values for the respective adducts (N_2_ as drift gas) [M + H]^+^: 325.25 Å^2^; [M-H]^−^: 332.58 Å^2^.

Suomilide C (**2**): white powder (4.1 mg); HRESIMS *m/z* 977.3781 [M – H]^−^ (calcd. for C_39_H_61_N_8_O_19_S, 977.3774) Mass error: 0.72 ppm. Retention Time_UPLC_: 2.10 min; CCS values for the respective adducts (N_2_ as drift gas) [M + H]^+^: 297.83 Å^2^; [M-H]^−^: 296.73 Å^2^.

Suomilide D (**3**): white powder (5.9 mg); HRESIMS *m/z* 1005.4089 [M – H]^−^ (calcd. for C_41_H_65_N_8_O_19_S, 1005.4087) Mass error: 0.20 ppm. Retention Time_UPLC_: 2.93 min; CCS values for the respective adducts (N_2_ as drift gas) [M + H]^+^: 309.25 Å^2^; [M-H]^−^: 308.06 Å^2^.

Suomilide E (**4**): white powder (2.6 mg); HRESIMS *m/z* 1047.4171 [M – H]^−^ (calcd. for C_43_H_67_N_8_O_20_S, 1047.4192) Mass error: 2.01 ppm. Retention Time_UPLC_: 3.30 min; CCS values for the respective adducts (N_2_ as drift gas) [M + H]^+^: 317.28 Å^2^; [M-H]^−^: 320.59 Å^2^.

#### 3.2.2. Structure elucidation

**Figure 3:**
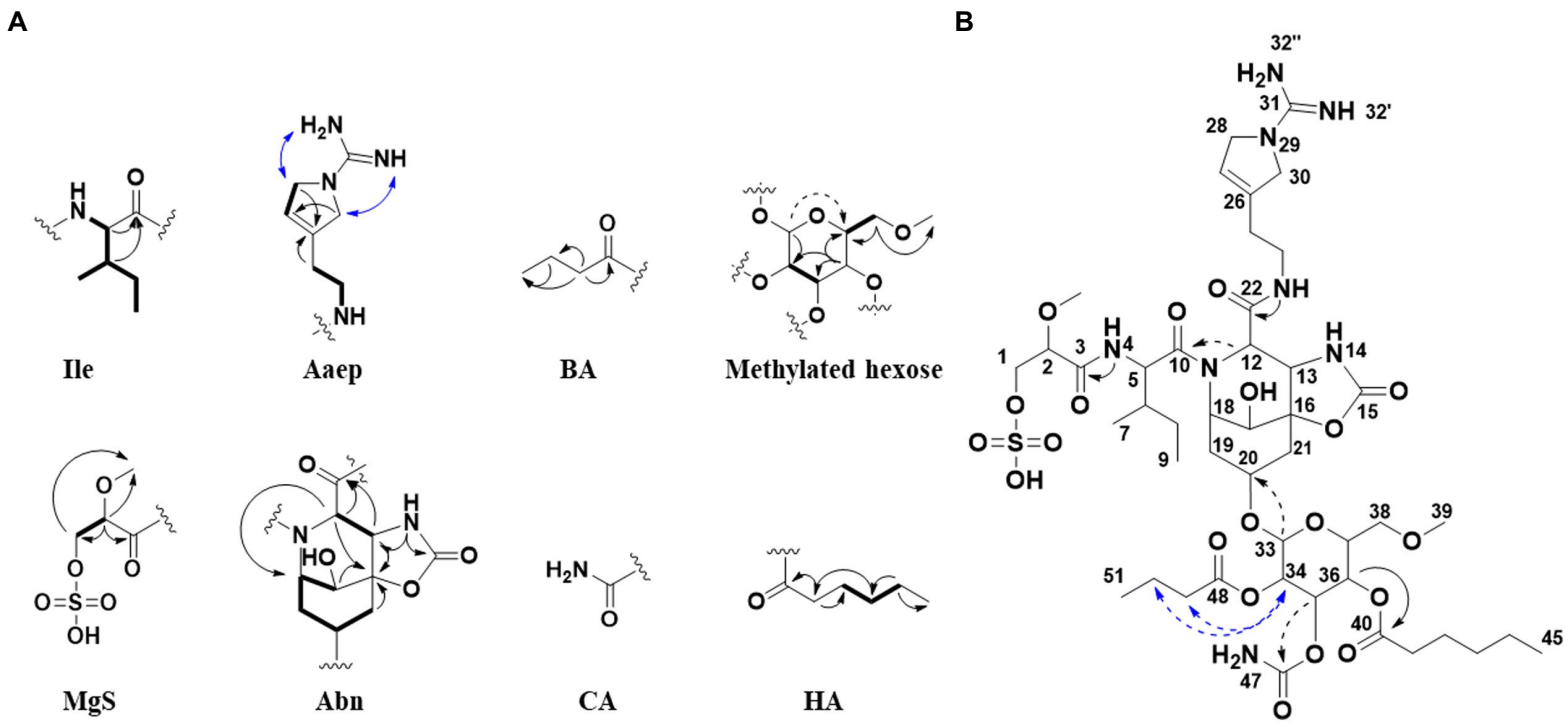
**(A)** Selected 2D NMR correlations obtained for substructures of **1**. **(B)** Key 2D NMR correlations used to connect the substructures and elucidate the complete structure of **1**. HMBC (black arrows), COSY (bold), ROESY (blue arrows). Weak correlations are indicated with dashed arrows. Ile, isoleucine; Aaep, 1-amidino-3-(2-aminoethyl)-3-pyrroline; BA, butyric acid; MgS, 2-O-methylglyceric acid 3-O-sulfate; Abn, azabicyclononane; CA, carbamic acid; HA, hexanoic acid.

Suomilide B (**1**) (Figure 1A) was isolated as white crystalline substance. The molecular formula was calculated to be C_45_H_72_N_8_O_20_S by HRESIMS, suggesting a presence of 14 degrees of unsaturation. 1D (^1^H and ^13^C, Tables 1, 2 and Supplementary Figures S4, S5) and 2D (HSQC, HMBC, COSY, ROESY, Supplementary Figures S6–S8) NMR data resembled those reported for **7** (Supplementary Table S9) and allowed seven substructures of **1** to be assigned. The substructures were isoleucine (Ile), 1-amidino-3-(2-aminoethyl)-3-pyrroline (Aaep), azabicyclononane (Abn), glycolipid with a methylated hexose core decorated with the subunits butyric acid (BA), carbamic acid (CA) and hexanoic acid (HA) (Figure 3A). An additional substructure, 2-O-methylglyceric acid 3-O-sulfate (MgS), was partially assigned, the sulfate group at C-1 was finally assigned based on elimination of every other possible binding site for the group (Figure 3B).

**Table 1:** ^1^H NMR assignments for suomilides B – E (**1**–**4**) (^1^H 600 MHz, DMSO-d_6_).

	δ_H_ (*J* in Hz)			
Position	1	2	3	4
1a	3.96–3.90, m*	3.97–3.85, m*	3.94, m	3.92, m*
1b	3.76, dt (11.9, 7.7)	3.76, dd (11.6, 7.8)	3.74, m	3.76, m*
2	3.96–3.90, m*	3.97–3.85, m*	3.92, m	3.92, m*
4	7.94, d (6.9)	8.00–7.92, m*	7.92, d (7.1)	7.88, d (7.2)
5	4.62, t (6.9)	4.62, t (7.0)	4.63, t (6.8)	4.63, t (7.0)
6	1.72, m*	1.71, m*	1.71, s*	1.70, m*
7	0.92–0.83, m*	0.92–0.79, m*	0.92–0.85, m*	0.96–0.78, m
8a	1.29, m*	1.29, m	1.29, m	1.29, m
8b	1.19, m	1.17, m	1.17, m	1.16, m
9	0.92–0.83, m*	0.92–0.79, m*	0.92–0.85, m*	0.96–0.78, m
12	4.52, d (2.4)	4.54, m	4.56, dd (7.4, 2.2)	4.52, d (2.4)
13	4.22, s	4.23, m	4.24, s*	4.20, d (2.4)
14	7.98, s	8.00–7.92, m*	8.10, s	8.09, s
17	3.72, s*	3.71, m	3.71, m	3.72, m*
18	4.28, s	4.27, m	4.24, s*	4.26, d (13.6)
19a	2.14, d (11.1, 5.4)	2.12, d (12.9)	2.15, d (11.8)	2.13, t (7.4)
19b	1.72, m*	1.71, m*	1.71, s*	1.72, m*
20	3.72, m*	3.69, m	3.72, m*	3.75, m*
21a	2.41–2.24, m*	2.30, m*	2.29, m*	2.32–2.23, m*
21b	1.99, dd (11.1, 5.4)	1.96, m	1.94, m	1.98, m
23	7.59, s	7.61, s	7.73, s	7.73, d (6.0)
24	3.18, m	3.18, m	3.19, m	3.19, m
25	2.41–2.24, m*	2.32–2.23, m*	2.26, m*	2.32–2.23, m*
27	5.64, s	5.64, s	5.63, s	5.64, s
28	4.17–4.11, m*	4.12, m	4.10, m*	4.12, m*
30	4.17–4.11, m*	4.12, m	4.11, m*	4.11, m*
32′/32″	7.25, s	7.23, s	7.20, s	7.66, s
33	4.97, d (3.8)	4.90–4.80, m	4.85, d (3.8)	4.96, d (3.8)
34	4.83, dd (11.0, 3.7)	4.90–4.80, m	4.49, dd (10.4, 3.7)	4.84, dd (11.0, 3.7)
35	5.02, dd (11.0, 3.5)	4.95, dd (10.7, 3.9)	4.95, dd (11.2, 8.4)	5.02, dd (11.0, 3.4)
36	5.34, d (3.3)	3.89, m	3.33, m*	5.35, m
37	4.17–4.11, m*	3.88, m	3.67, m	4.14, m
38a	3.34, m	3.44, m	3.50, m	3.34, m
38b	3.25, dd (10.0, 6.0)	3.38, m	3.38, m*	3.25, m
39	3.21, s	3.25, s	3.25, s	3.21, s
41	2.41–2.24, m*	–	2.27, m*	2.32–2.23, m*
42	1.54, m*	–	1.51, *p* (7.3)	1.60–1.49, m*
43	1.28, m*	–	1.24, m*	0.96–0.78, m
44	1.28, m*	–	1.26, m*	–
45	0.92–0.83, m*	–	0.92–0.85, m*	–
47	6.56, s	6.50, s	6.44, m	6.56, s
49	2.41–2.24, m*	2.32–2.23, m*	–	2.32–2.23, m*
50	1.54, m*	1.53, m	–	1.60–1.49, m*
51	0.92–0.83, m*	0.92–0.79 (m)*	–	0.96–0.78, m
2me	3.30, s	3.30, s	3.30, s	3.30, s

* Peaks are overlapped; n.d., not detected; −, protons not part of the structure.

**Table 2:** ^13^C NMR assignments for suomilides B – E (**1**–**4**) (^13^C 150 MHz, DMSO-d_6_).

	δ_C_, type					δ_C_, type			
Position	1	2	3	4	Position	1	2	3	4
1	66.2, CH_2_	65.9, CH_2_	66.2, CH_2_	66.2, CH_2_	28	55.4, CH	55.4, CH	55.3, CH_2_	55.3, CH_2_
2	80.2, CH	79.9, CH	80.3 CH	80.3, CH	30	54.2, CH	54.2, CH	54.1, CH_2_	54.1, CH_2_
3	169.8, C	169.7, C	169.7, C	169.7, C	31	154.2, C	154.0, C	154.5, C	154.5, C
5	53.1, CH	52.9, CH	53.0, CH	52.9, CH	33	94.7, CH	94.6, CH	94.2, CH	94.0, CH
6	36.5, CH	36.3, CH	36.5, CH	36.5, CH	34	68.0, CH	68.8, CH	71.0, CH	68.0, CH
7	14.5, CH_3_	14.1, CH_3_	14.5, CH_3_	14.5, CH_3_	35	66.6, CH	67.7, CH	71.5, CH	66.6, CH
8	25.4, CH_2_	25.3, CH_2_	25.5, CH_2_	25.4, CH_2_	36	68.4, CH	66.4, CH	68.0, CH	68.4, CH
9	11.8, CH_3_	11.5, CH_3_	11.8, CH_3_	11.8, CH_3_	37	67.0, CH	68.7, CH	71.4, CH	67.0, CH
10	171.9, C	171.2, C	171.8, C	171.8, C	38	70.0, CH_2_	70.4, CH_2_	70.8, CH_2_	70.0, CH_2_
12	56.9, CH	56.6, CH	56.8, CH	57.0, CH	39	58.5, CH_3_	58.4, CH_3_	58.5, CH_3_	58.6, CH_3_
13	58.3, CH	57.9, CH	58.2, CH	58.2, CH	40	172.0, C	–	172.6, C	171.8, C
15	156.7, C	156.7, C	156.6, C	156.7, C	41	33.3, CH_2_	–	33.5, CH_2_	35.3, CH_2_
16	80.5, C	80.3, C	80.5, C	80.5, C	42	24.1, CH_2_	–	24.1, CH_2_	18.0, CH_2_
17	65.7, CH	65.6, CH	65.8, CH	65.7, CH	43	30.6, CH_2_	–	30.5, CH_2_	13.4, CH_3_*
18	53.4, CH	53.2, CH	53.5, CH	53.4, CH	44	21.8, CH_2_	–	21.8, CH_2_	-
19	28.8, CH_2_	28.6, CH_2_	28.8, CH_2_	28.9, CH_2_	45	13.8, CH_3_	–	13.8, CH_3_	–
20	70.0, CH	69.1, CH	69.3, CH	70.0, CH	46	155.4, C	156.3, C	156.3, C	155.4, C
21	34.4, CH_2_	34.4, CH_2_	34.4, CH_2_	34.5, CH_2_	48	172.6, C	172.3, C	–	172.6, C
22	168.8, C	168.7, C	168.8, C	168.9, C	49	35.3, CH_2_	35.2, CH_2_	–	35.5, CH_2_
24	37.2, CH_2_	36.9, CH_2_	37.2, CH_2_	37.2, CH_2_	50	18.0, CH_2_	17.8, CH_2_	–	18.0, CH_2_
25	27.8, CH_2_	27.6, CH_2_	27.8, CH_2_	27.8, CH_2_	51	13.4, CH_3_	14.1, CH_3_	–	13.4, CH_3_*
26	135.9, C	135.9, C	136.0, C	136.0, C	2me	57.3, CH_3_	57.07, CH_3_	57.3, CH_3_	57.4, CH_3_
27	119.0, CH	118.8, CH	119.0, CH	119.0, CH					

*Peaks are overlapped; −, carbon atoms not part of the structure.

The Abn substructure was assigned based on COSY and HMBC correlations, and by comparing our data to previously published data (Fujii et al., 1997). A COSY spin system was observed from H-17 (δH 3.72) to H-21a (δH 2.14)/H-21b (δH 1.99). The shift value of the tertiary C-17 (δC 65.7) suggested hydroxylation in this position. HMBC correlations furthermore linked both H-17 and H-21a to the quaternary C-16 (δC 80.5) carbon atom, which was further linked to H-13 (δH 4.22) through an HMBC correlation. The downfield shift value of C-16 (δC 80.5) suggested that it was linked to an oxygen. H-13 was linked to H-12 (δH 4.52) through a COSY correlation. A HMBC correlation was observed between NH-14 (δH 7.98) and carbon atoms C-13 (δC 58.3) and C-15 (δC 156.7). The de-shielded shift value of C-15 (δC 156.7) was characteristic for a carboxyl carbon, placing an oxygen atom at this position, and attached C-15 to C-16 via an ester linkage. Our 1D NMR data closely resembles that of the previously published data for the Abn subunit. When comparing 1D NMR data for protons and carbons 12–22 to the same data recorded for **7** (Fujii et al., 1997), the ΔδC shift values varies on average 0.12 ppm and ΔδH shift values varies on average 0.03 ppm (data recorded in DMSO-d_6_, Supplementary Table S9). This confirmed that **1** had the Abn moiety, which is a collective feature of the bsl family of compounds.

The Ile subunit was assigned based on typical proton and carbon chemical shifts and correlations in HMBC and COSY spectra and was found to be attached from C-10 (δC 171.9) to position 11 of the Azbn subunit through a weak HMBC correlation between H-12 (δH 4.52) and C-10 (δC 171.9). This places a nitrogen in the 11 position and completes the tricyclic Abn subunit. The MgS subunit was assigned based on 1D NMR shift values and HMBC and COSY correlations. It was found to be attached to N-4 of the Ile group through an HMBC correlation between NH-4 (δH 7.94) and C-3 (δC 169.8). A sulfate group at C-1 based on elimination of every other possible binding site for the group. The glycosyl group of the glycolipid subunit was determined to be methylated hexose based on typical proton and carbon chemical shifts and correlations in HMBC and COSY spectra. The hexose was determined to be methylated through an HMBC correlation between H-38a (δH 3.34) and H-38b (δH 3.25) and the primary carbon atom C-39 (δC 58.5). The glucose subunit was found to be attached through an ether bond to C-20 of the Abn subunit through a weak HMBC correlation between H-33 (δH 4.97) and C-20 (δC 70.0). The CA subunit was assigned based on 1D and 2D NMR data and was found to be linked to the hexose subunit through an ester bond determined by a weak HMBC correlation between H-35 (δH 5.02) and the quaternary C-46 carbon atom (δC 115.4). The HA and BA subunits were identified by correlations in the HMBC and COSY spectra. The HA and BA subunits were linked to the hexose subunit through ester bonds. The HA subunit was placed at C-36-O through an HMBC correlation between H-36 (δH 5.34) and C-40 (δC 172.9). The BA subunit was found to be linked to C-34 through an ether bond through weak ROESY correlations between H-34 (δH 4.83) and the BA protons H-49 (δH 2.41–2.24) and H-50 (δH 1.54). Consequently, the structure of **1** was assigned.

Suomilide C (**2**) (Figure 1A) was isolated as white crystalline substance. The molecular formula was calculated to be C_39_H_62_N_8_O_19_S by HRESIMS, suggesting a presence of 13 degrees of unsaturation. The mass and elemental composition of **2** indicated that its structure was closely related to that of **1**. By close inspection of 1D (^1^H, ^13^C, Tables 1, 2 and Supplementary Figures S10, S11) and 2D (HSQC, HMBC, COSY, TOCSY and ROESY, Supplementary Figures S12–S15) NMR data, the structure of 2 was elucidated in a similar manner as described above for **1**. In the ^13^C spectra, only 22 of the carbon atoms gave prominent peaks. The remaining carbon atom shift values were extracted from the HSQC spectra. When comparing the ^1^H and ^13^C chemical shift values of **1** and **2** for the MgS, Ile, Abn, Aaep, CA and BA substructures, the values were found to conform well (ΔδC average: 0.2 ppm, ΔδH average: 0.013 ppm). The most noticeable difference between the ^1^H-NMR datasets of **1** and **2**, was the lack of a proton resonance for H-36 at 5.34 ppm in the ^1^H spectrum of **2**. Instead, H-36 was found to have a shift value of 3.86 ppm. The shift value of C-36 had also changed from 68.4 ppm in **1** to 66.4 in **2**. The shielding of CH-36 could be explained by elimination of the HA subunit, causing C-36 to be attached to a hydroxyl group rather than, as in **1**, an ester. Elimination of HA was in line with the difference in the calculated elemental compositions of **1** and **2**. Signals from the HA subunit were however still visible but were significantly less prominent in the spectra recorded for **2**. Thus, the structure of **2** was confirmed and **1** was confirmed to be present in the sample of **2** as a minor component.

Suomilide D (**3**) (Figure 1A) was isolated as white crystalline substance. The molecular formula was calculated to be C_41_H_66_N_8_O_19_S by HRESIMS, suggesting a presence of 13 degrees of unsaturation. Compared to **1**, the calculated elemental composition of **3** indicated the compound as a variant of **1** lacking BA on the methylated hexose subunit. 1D (^1^H and ^13^C, Tables 1, 2 and Supplementary Figures S16, S17) and 2D (HSQC, HMBC, COSY and ROESY, Supplementary Figures S18–S20) NMR analysis, confirmed that **3** consisted of the Ile, Abn, Aaep, CA and HA subunits. The positions of the Ile, Abn and Aaep subunits were confirmed in a similar manner as described for **1**. In the COSY spectrum (Supplementary Figure S19), H-36 (δH 3.36) coupled to a hydrogen atom at 5.32 ppm. This shift value is comparable to the shift values for the hydroxyl hydrogens on the hexosesubstructure of suomilide (δH 36-OH 5.23, δH 35-OH 5.31) (Fujii et al., 1997), confirming that **3** had an unsubstituted hydroxyl group on C-36 (δC 68.0). The CA subunit was linked to C-35 (δC 71.3) through a HMBC correlation from H-35 (δH 4.96) to C-46 (δC 156.3) (Supplementary Figure S22). The HA subunit was linked C-34 (δC 70.8) through a weak ROESY correlation between H-34 (δH 4.48) and H-41 (δH 2.27) (Supplementary Figure S20). Finally, the structure of the MgS subunit was determined in a similar manner as described for **1**, and the sulfate group was placed at C-1 (δC 65.9) after elimination of every other possible binding site for the group.

Suomilide E (**4**) (Figure 1A) was isolated as white crystalline substance. The molecular formula was calculated to be C_43_H_68_N_8_O_20_S by HRESIMS, suggesting a presence of 14 degrees of unsaturation. The structure of **4** was assigned by 1D (^1^H and ^13^C, Tables 1, 2 and Supplementary Figures S21, S22) and 2D (HSQC, HMBC, COSY, TOCSY, ROESY and HSQC-HSQCTOCSY, Supplementary Figures S23–S28) NMR experiments. In a similar matter as described above, **4** was confirmed to contain the MgS, Ile, Abn, Aaep and methylated hexose subunits. The decoration of the methylated hexose was determined to be two BA subunits and a CA subunit. One BA subunit was attached to C-34 (δC 68.0) through a HMBC between H-34 (δH 4.84) to C-48. The second BA subunit was placed at C-36 (δC 66.6) through a HMBC between H-36 (δH 5.35) to C-40 (δC 171.8). The placement of the CA subunit was, as for **1**–**3**, determined to be at C-35 (δC 66.6) through a weak HMBC between H-35 (δH 5.02) and C-46 (δC 155.4). The sulfate group placed at C-1 (δC 66.2) after elimination of every other possible binding site for the group. Thus, the structure of **4** was elucidated.

### 3.3. Biological evaluation of compounds 1–4

With the isolated compounds **1**–**4** at hand, it was possible to investigate the bioactivity of all four compounds. Since the production of secondary metabolites represents a metabolic and energetic effort, they are likely to give a selective advantage to the producing organism (Maplestone et al., 1992) or have a function within the organism. Were therefore tested to see if **1**–**4** had any effect on the survival of bacterial cells as well as on formation of bacterial biofilm. In addition, the compounds were screened for potential anti-proliferative effects on malignant and non-malignant human cells. We also wanted to investigate if the previously observed bioactivity of this strain (Liaimer et al., 2016) is related to the isolated suomilides, by assaying the ability of **1**–**4** to act as protease inhibitors. For the bioassays, **1**–**4** were dissolved in DMSO and further diluted in ddH_2_O.

#### 3.3.1. Antibacterial and antibiofilm activity

There were no significant effects of **1**–**4** when tested at concentrations up to 100 μM against *Staphylococcus aureus*, *Escherichia coli*, methicillin resistant *S. aureus*, *Pseudomonas aeruginosa*, *Enterococcus faecalis* and *Streptococcus agalactiae*. There were also no effects on biofilm formation by *Staphylococcus epidermidis* at concentrations up to 100 μM.

#### 3.3.2. Cytotoxicity against malignant and non-malignant cell lines

The crude extract of KVJ20 was initially assayed against a panel of human cell lines showing anti-proliferative effects against the human non-malignant cell line MRC5 (lung fibroblast) and the human malignant cell line A2058 (melanoma), but the previous study did not show that the extract had any effect against the human malignant cell line HT29 (colon carcinoma) (Liaimer et al., 2016). Therefore, we investigated the bioactivity of **1**–**4** against MRC5 and A2058 as well as the human malignant cell line MOLM13 (acute myeloid leukemia). Compounds **1**–**4** were assayed at concentrations up to 100 μM. No effects were observed.

Compounds 5 and 6 were originally isolated via bioassay guided purification using a serine-protease inhibition assay when they were discovered in 2005 (Pluotno and Carmeli, 2005). The two banyasides were reported to inhibit the catalytic activity of trypsin. As far as we know, anti-bacterial activity of suomilide have not been reported previously, and the *Nostoc* sp. strain it has been isolated from was reported as non-toxic. This complies with our results, as no activity could be detected for **1**–**4** against bacteria or cell lines at high concentrations. Suomilide A has recently been investigated for serine protease inhibition and has been shown to inhibit trypsin-1, −2 and − 3 with IC_50_ values of 104, 4.7 and 11.5 nM, respectively (Ahmed et al., 2021). Molecular docking studies of suomilide A revealed that the Aeap and Mgs moieties are responsible for the compound-target interaction with trypsine, confirmed by surface plasmon resonance spectroscopy the revealing residence time of 57 min for trypsin-3 was determined (Ahmed et al., 2021). A concentration of 3.3 μM of suomilide A was shown to inhibit the invasion of prostate cancer cells in a cell invasion assay while it had no effect on cancer cell proliferation (Ahmed et al., 2021), which is in accordance with our results. Taking a closer look on the structure of other cyanobacterial protease inhibitors, such as cyanopeptolins, microviridins and others, it appears that they are cyclic peptides in contrast to the rigid modified core of the suomilides and banyasides (Singh et al., 2011; Gallegos et al., 2018; Mazur-Marzec et al., 2018; Sieber et al., 2020). The suomilides on the other hand clearly belong to the aeruginosin family of protease inhibitors (Ersmark et al., 2008).

### 3.4. Biosynthesis of the suomilides

**Figure 4:**
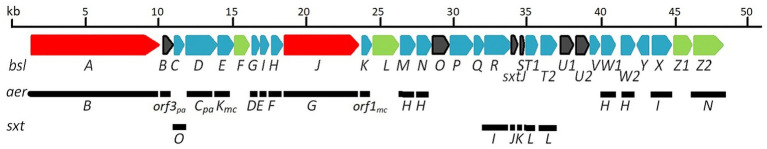
*bsl*-biosynthetic cluster proposed for suomilides: At the top the *bsl*-gene cluster. Below the indication of which genes are similar to those in the respective gene clusters of aeruginosin (*aer*) and saxitoxin (*sxt*). A detailed description is given in Supplementary Table S29. NRPS genes are given in red, other biosynthetic genes in blue and transporters/transport related genes in green. Open reading frames/hypothetical genes are colored black. Genes found only in *Microcystis aeruginosa* or *Planctothrix aghardii* are marked with _mc_ and _pa_, respectively.

A previous study predicted the presence of 19 gene clusters in KVJ20 containing genes involved in the biosynthesis of nonribosomal peptides, polyketides, and ribosomally synthesized and posttranslational modified peptides (Halsør et al., 2019). In addition to the well-defined anabaenopeptin and nostocyclopeptide gene clusters, we were able to identify genes associated with aeruginosin production and assemble the entire *bsl* gene cluster. The reassembled *bsl* cluster can be retrieved under the gene bank accession number: MT269816 (Figure 4). The cluster consists predominantly of genes that are also present in aeruginosin and saxitoxin (molecular structure in Figure 2) gene clusters. For aeruginosin, the most similar clusters are aeruginosin 126B (BGC0000297) where 41% of the genes show similarity and aeruginosin 98-A (BGC0000298, 42% of genes show similarity).

**Figure 5:**
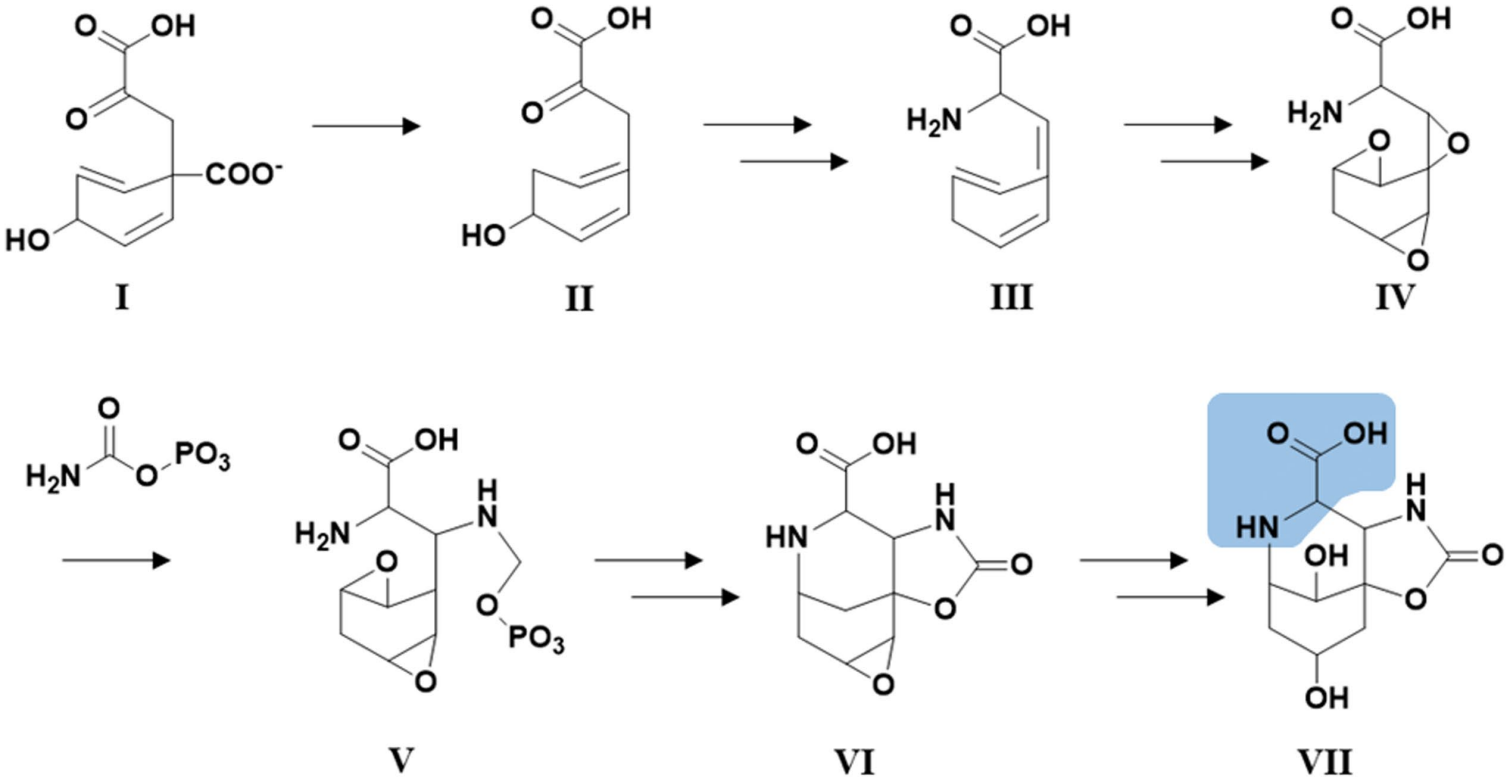
Hypothetical biosynthetic pathway for the Abn moiety (VII) based on Pluotno and Carmeli (2005) but starting from prephenate (I). Structural feature highlighted in blue: The structural features of VII which are distantly similar to proline.

For saxitoxin, the clusters BGC0000887, BGC0000188 and BGC0000928 show a similarity of 14%. The genes and the respective clusters they originate from are given in Supplementary Table S32 and illustrated in Figure 4. We propose the cluster described here (Figure 4 and Supplementary Table S32) is the biosynthetic gene cluster responsible for the production of the suomilides. The proposed functions for the respective genes are given in Supplementary Table S32. For the banyasides, the biosynthesis of the Abn moiety was proposed to start from l-tyrosine (Pluotno and Carmeli, 2005). The present cluster, however, possesses prephenate decarboxylase (*bslG*), as predicted via anti-smash. We therefore propose an alternative biosynthesis starting from prephenate instead of tyrosine as shown in Figure 5. The biosynthesis of secondary metabolites from prephenate involving prephenate decarboxylases has been observed for bacilysin, salinosporamide A and aeruginoside 126A as well (Mahlstedt et al., 2010). Ahmed et al. (2021) suggested the synthesis of Abn from the Choi moiety (highlighted in Figure 2), starting off from prephenate as well and proposed a similar cluster. They have also identified the presence of the *sxtJ* and *sxtK* genes (see Ahmed et al., 2021; Supplementary Table S2). We have identified *sxtJ,K,L* and *O*-like genes (see Figure 4 and Supplementary Table S29). Another group of protease inhibiting natural products bearing the 2-carboxyl-6-hydroxyoctahydroindole (Choi) moiety are the dysinosins (see Figure 2) that were originally isolated from sponges but are likely to be produced by a cyanobacterial symbiont (Carroll et al., 2002, 2004; Schorn et al., 2019). Also Ahmed et al. compared their cluster, among others, to those of Dysinosin B and Aeruginoside 126A which are related. Based on our findings we hypothesize that the Suomilide BGC shows genes that originate from two “parent” BGCs, one saxitoxin-like and one aeruginoside-like. Interestingly, within our assembly, the genes *sxtJ,K* and *L* cluster together within the BGC as well. The gene *bslJ* is coding a NRPS subunit predicted to incorporate isoleucine which is apparently present in the suomilides. However, *bslA* is predicted to code for a NRPS incorporating proline, we hypothesize that the NRPS-subunit is binding the Abn moiety due to its distant structural similarity to proline (see Figure 5, VII). The proposed cluster and its genes can be related to the structural properties of suomilides.

**Figure 6:**
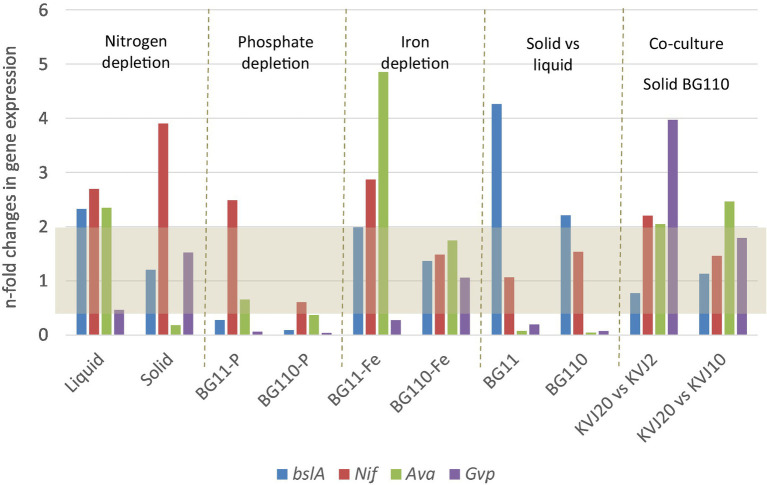
Results of the gene expression studies. The results are given as gene expression n-fold change in *Nostoc* sp. KVJ20 for the NRPS gene *bslA*, marker for nitrogen fixation nitrogenase (*nifH*), akinete formation marker (*avaK*), and hormogonia differentiation marker (*gvpC*). The fold changes were calculated as follows. For nitrogen depletion liquid cultures in BG110 medium were compared to liquid cultures in BG11 medium; similarly, the cultures on solid BG110 were compared to cultures on solid BG11. For iron and phosphate depletions, as well as solid vs. liquid grown, the nitrogen regime was the same in mutually compared cultures. The gene expression in colonies grown aside another strain (co-culture) on BG110 agar were compared to cultures on solid BG110 medium with another *Nostoc* sp. KVJ10 or KVJ2 colony as neighbor (Liaimer et al., 2016). The gray area covers the values with less than two-fold change in expression.

Potential ecological functions of the suomilides may be related to their serine-protease inhibition, such as anti-grazing activity (Ferrão-Filho and Kozlowsky-Suzuki, 2011; Sieber et al., 2020). This is supported by the fact that the bacteria accumulate suomilides within the cells, and do not release notable amounts to the growth media (Liaimer et al., 2016). In a gene-expression study we investigated the expression of the *bslA* gene and differentiation marker genes under phosphate, iron and nitrogen depletion as well as solid/liquid media. Gene expression patterns in presence of two other cyanobacterial strains (KVJ2 and KVJ10) were also investigated. *bsl* genes showed higher relative expression levels in the cultures under nitrogen limitation. No up-regulation in response to phosphate and iron removal was observed, neither did the presence of competitor strains induce higher transcript levels (see Figure 6 and for a detailed discussion of the results of the gene-expression studies Supplementary material S30). Therefore, it is feasible to suggest that suomilides are in one way or another related to the diazotrophic growth of the producer strain.

## 4. Conclusion

We were able to isolate four suomilide variants and to elucidate their structures. The compounds did not show any bioactivity against bacteria, bacterial biofilm-generation or against human cell lines, which is in accordance with previous studies demonstrating that suomilide A stops the infiltration of prostate cancer cells but not their proliferation. The suomilides A-E differ among each other in the decoration of their glycon. The biosynthetic gene-cluster we propose for the suomilides suggests that the biosynthesis of azabizyclononane starts from prephenate and the cluster comprises genes from both, aeruginosin and saxitoxin gene clusters.

## Data availability statement

The datasets presented in this study can be found in online repositories. The names of the repository/repositories and accession number(s) can be found in the article/Supplementary material.

## Author contributions

YS: extraction, isolation, bioactivity testing, HPLC-MS analysis, and preparation of the manuscript. AL and OW: dereplication, cultivation of biomass, genome mining, molecular biology, and preparation of the manuscript. KH: structure elucidation and preparation of the manuscript. JI: NMR spectroscopy and structure elucidation. JA and EH: preparation of the manuscript and review. All authors contributed to the article and approved the submitted version.

## Funding

This project was received from the Marie Skłodowska-Curie Action MarPipe, grant agreement GA 721421 H2020-MSCA-ITN-2016, and from UiT – The Arctic University of Norway.

## Conflict of interest

The authors declare that the research was conducted in the absence of any commercial or financial relationships that could be construed as a potential conflict of interest.

## Publisher’s note

All claims expressed in this article are solely those of the authors and do not necessarily represent those of their affiliated organizations, or those of the publisher, the editors and the reviewers. Any product that may be evaluated in this article, or claim that may be made by its manufacturer, is not guaranteed or endorsed by the publisher.
