# Corrigendum: Four new suomilides isolated from the cyanobacterium *Nostoc* sp. KVJ20 and proposal of their biosynthetic origin

**DOI:** 10.3389/fmicb.2023.1221368

**Published:** 2023-06-07

**Authors:** Yannik K.-H. Schneider, Anton Liaimer, Johan Isaksson, Oda S. B. Wilhelmsen, Jeanette H. Andersen, Kine Ø. Hansen, Espen H. Hansen

**Affiliations:** ^1^Marbio, Faculty of Biosciences, Fisheries and Economics, UiT—The Arctic University of Norway, Tromsø, Norway; ^2^Department of Arctic and Marine Biology, Faculty of Biosciences, Fisheries and Economics, UiT—The Arctic University of Norway, Tromsø, Norway; ^3^Department of Chemistry, Faculty of Natural Sciences, UiT—The Arctic University of Norway, Tromsø, Norway

**Keywords:** *Nostoc*, cyanobacteria, natural products, protease inhibitor, biosynthesis, secondary metabolites, aeruginosin, suomilide

In the published article, there was an error in Figure 1 as published. In Figure 1, the list of compounds was incorrectly presented, such that six compounds were listed rather than seven and the order of compounds was not correct. The corrected Figure 1 and its caption appear below.

**Figure 1:**
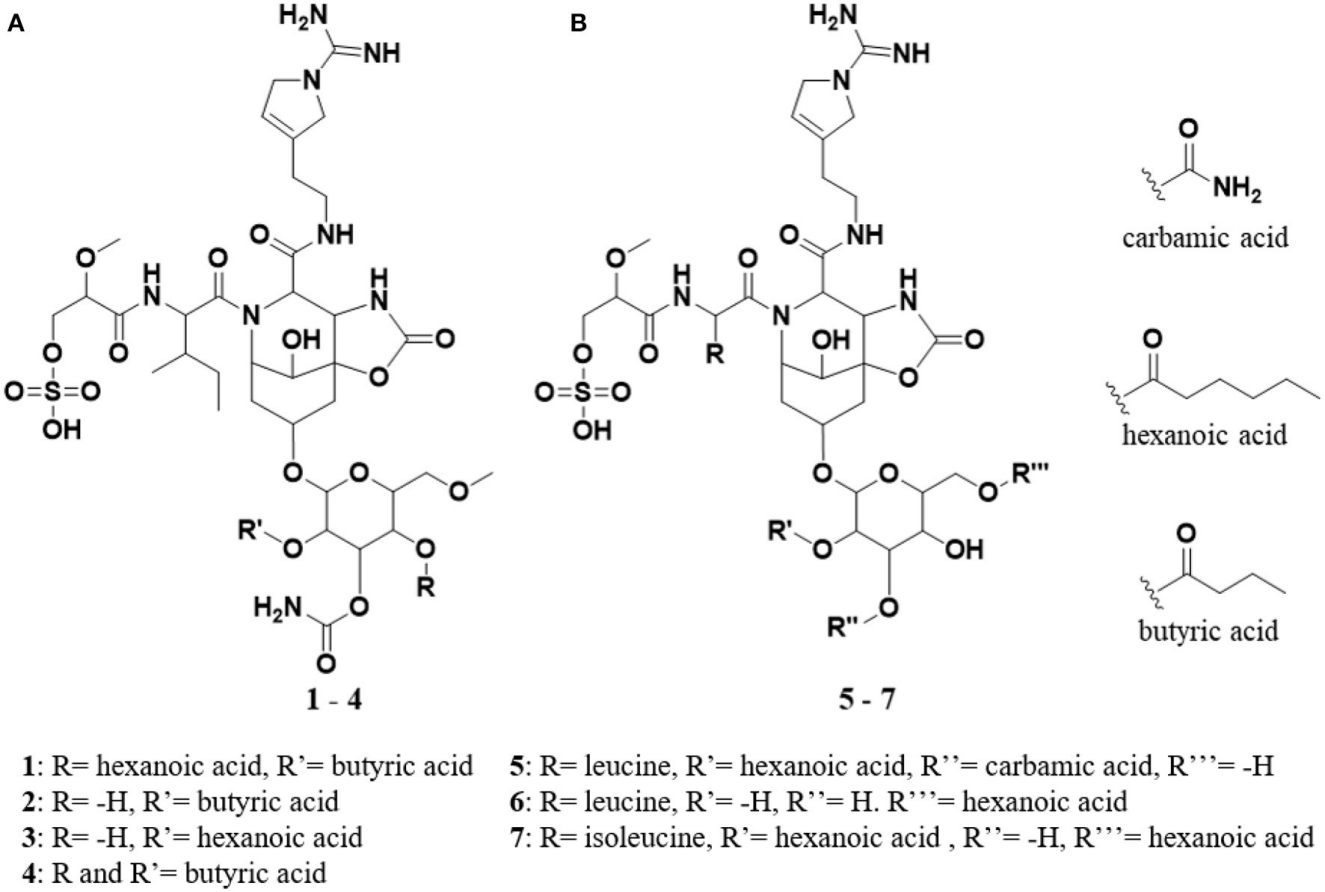
**(A)** Keep bold structures of suomilide B – E (**1–4**). **(B)** The previously isolated molecules banyasides A and B (**5** and **6**) and suomilide (**7**). All molecules share an Abn (azobicyclononane) core and an Aeap-moiety [1-amino-2-(N-amidino-Δ3-pyrrolinyl)ethyl], which also can be observed in the aeruginosins, as well as leucine and glycosylation. Suomilide differs from the banyasides by incorporation of isoleucine instead of leucine. The banyasides differ in the modification of their glycons (α-glucose for **4**, **5**, and **6**.

The authors apologize for this error and state that this does not change the scientific conclusions of the article in any way. The original article has been updated.

