# Supplementary material for: Four new suomilides isolated from the cyanobacterium Nostoc sp. KVJ20 and proposal of their biosynthetic origin
Source: Front Microbiol. 2023 Apr 20;14:1130018. doi: 10.3389/fmicb.2023.1130018 (PMC10157211; doi:10.3389/fmicb.2023.1130018)
Supplement: Supplementary file 1 [file Data_Sheet_1.pdf]

Supplementary Materials:

Four new Suomilides isolated from the Cyanobacterium *Nostoc* sp. KVJ20 and Proposal of their Biosynthetic Origin

Yannik K.-H. Schneider[1], Anton Liaimer[2], Johan Isaksson[3], Oda S. B. Wilhelmsen[2], Jeanette H. Andersen[1], Kine Ø. Hansen[1], and Espen H. Hansen[1]*

[1] Marbio, Faculty for Fisheries, Biosciences and Economy, UiT—The Arctic University of Norway, N-9037 Tromsø, Norway.

[2] Department of Arctic and Marine Biology, Faculty for Fisheries, Biosciences and Economy, UiT—The Arctic University of Norway, N-9037 Tromsø, Norway.

[3] Department of Chemistry, Faculty of Natural Sciences, UiT—The Arctic University of Norway, N-9037 Tromsø, Norway.

[*]Corresponding Author: yannik.k.schneider@uit.no

Table of Contents:

Mass-spectrometric data for dereplication

Figure S1.1 ESI$^+$-IMS-MS spectra of suomilide C

Figure S1.2 ESI$^-$-IMS-MS spectra of suomilide B

Figure S1.3 ESI$^-$-IMS-MS spectra of suomilide C

Figure S1.4 ESI$^-$-IMS-MS spectra of suomilide D

Figure S1.5 ESI$^-$-IMS-MS spectra of suomilide E

Figure S1.6 Proposed structure of fragment 610

Figure S2 ESI$^+$-IMS-MS identification of putative schizopeptin 791

Figure S3 Putative identification of schizopeptin 791

NMR Spectroscopic Data for suomilide B (1)

Figure S4 ^{1}H NMR (600 MHz, DMSO-d_6) spectrum of suomilide B (**1**)

Figure S5 ^{13}C (151 MHz, DMSO-d_6) spectrum of suomilide B (**1**)

Figure S6 HSQC + HMBC (600 MHz, DMSO-d_6) spectrum of suomilide B (**1**)

Figure S7 COSY (600 MHz, DMSO-d_6) spectrum of suomilide B (**1**)

Figure S8 ROESY (600 MHz, DMSO-d_6) spectrum of suomilide B (**1**)

Table S9 Comparison of 1D NMR chemical shift values between the azabicyclononane substructures of suomilide B (**1**) and suomilide (**7**)

NMR Spectroscopic Data for suomilide C (2)

Figure S10 ^{1}H NMR (600 MHz, DMSO-d_6) spectrum of suomilide C (**2**)

Figure S11 ^{13}C (151 MHz, DMSO-d_6) spectrum of suomilide C (**2**)

Figure S12 HSQC + HMBC (600 MHz, DMSO-d_6) spectrum of suomilide C (**2**)

Figure S13 COSY (600 MHz, DMSO-d_6) spectrum of suomilide C (**2**)

Figure S14 TOCSY (600 MHz, DMSO-d_6) spectrum of suomilide C (**2**)

Figure S15 ROESY (600 MHz, DMSO-d_6) spectrum of suomilide C (**2**)

NMR Spectroscopic Data for suomilide D (3)

Figure S16 ^{1}H NMR (600 MHz, DMSO-d_6) spectrum of suomilide D (**3**)

Figure S17 ^{13}C (151 MHz, DMSO-d_6) spectrum of suomilide D (**3**)

Figure S18 HSQC + HMBC (600 MHz, DMSO-d_6) spectrum of suomilide D (**3**)

Figure S19 COSY (600 MHz, DMSO-d_6) spectrum of suomilide D (**3**)

Figure S20 ROESY (600 MHz, DMSO-d_6) spectrum of suomilide D (**3**)

NMR Spectroscopic Data for suomilide E (4)

Figure S21 ^{1}H NMR (600 MHz, DMSO-d_6) spectrum of suomilide E (**4**)

Figure S22 ^{13}C (151 MHz, DMSO-d_6) spectrum of suomilide E (**4**)

Figure S23 HSQC + HMBC (600 MHz, DMSO-d_6) spectrum of suomilide E (**4**)

Figure S24 HSQC + HMBC (600 MHz, DMSO-d_6) spectrum highlighting selected correlations used to assign suomilide E (**4**)

Figure S25 COSY (600 MHz, DMSO-d_6) spectrum of suomilide E (**4**)

Figure S26 TOCSY (600 MHz, DMSO-d_6) spectrum of suomilide E (**4**)

Figure S27 ROESY (600 MHz, DMSO-d_6) spectrum of suomilide E (**4**)

Figure S28 HSQC + HSQCTOCSY (600 MHz, DMSO-d_6) spectrum highlighting selected correlations used to assign suomilide E (**4**)

Genomic data and gene-expression study:

Table S29 Genes of the identified *bsl*-cluster

Section S30 Gene expression study of BGC in *Nostoc* sp. KVJ20 subjected to different growth conditions

Table S31.1 Primers designed for *Nostoc* sp. KVJ20

Table S31.2 Morphological appearance of the Nostoc sp. KVJ20 subjected to different cultivation conditions.

Table S31.3 Changes in expression of genes for KVJ20 under different cultivation conditions

Literature

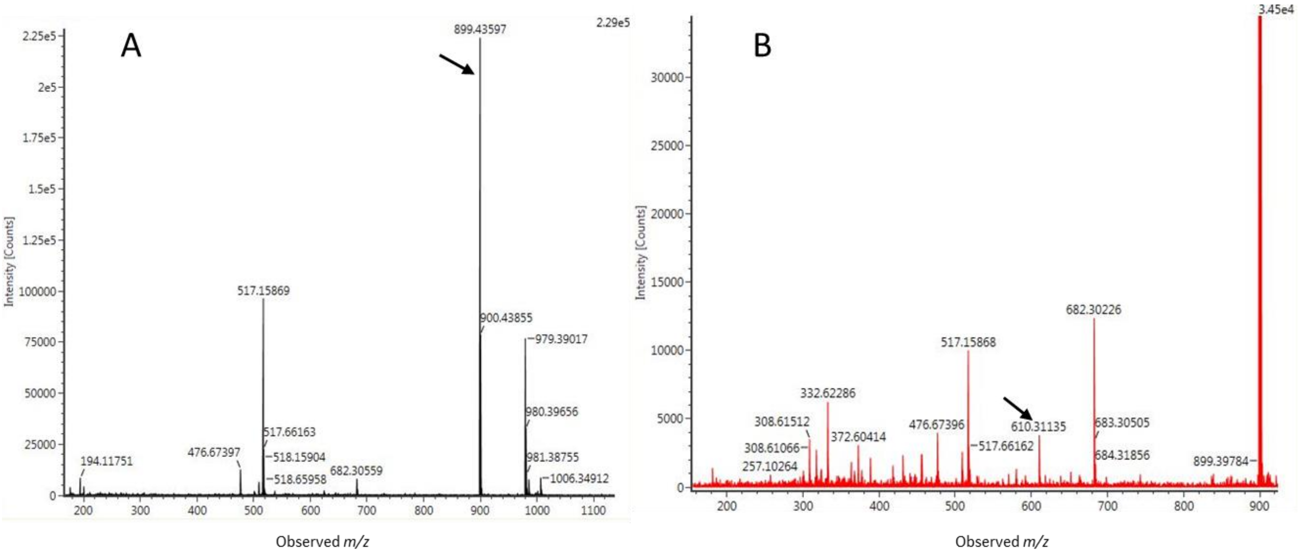

S1.1 ESI$^+$-IMS-MS spectra of suomilide C. A: Low- energy collision spectrum, the neutral loss of sulphate is indicated by the black arrow. B: High-energy collision spectrum (20-60 eV ramp) in red. The fragment at *m/z* 610.3206 is the desulfo-aglycon after hydrolytic loss of the sulfate and sugar-moiety, see **S1.6**. *m/z* 899.4340 is **2** after loss of sulfate.

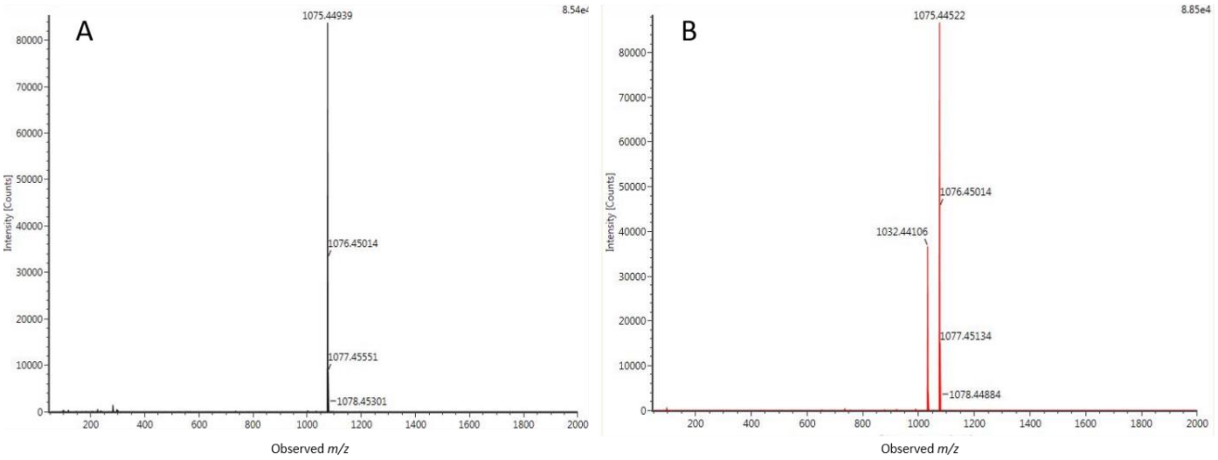

S1.2 ESI$^-$-IMS-MS spectra of suomilide B. A: Low- energy collision spectrum; B: High-energy collision spectrum (20-60 eV ramp) in red.

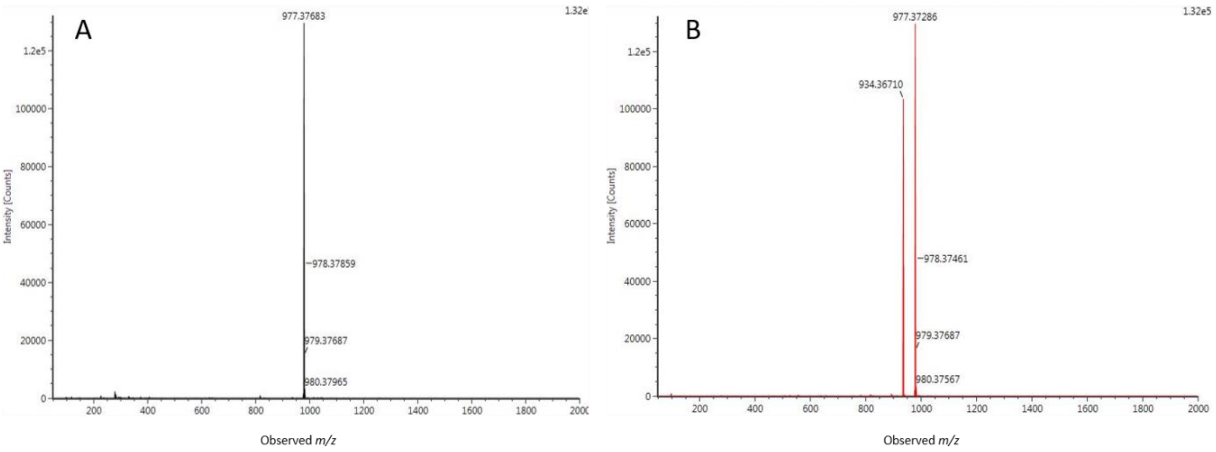

S1.3 ESI$^-$-IMS-MS spectra of suomilide C. A: Low- energy collision spectrum; B: High-energy collision spectrum (20-60 eV ramp) in red.

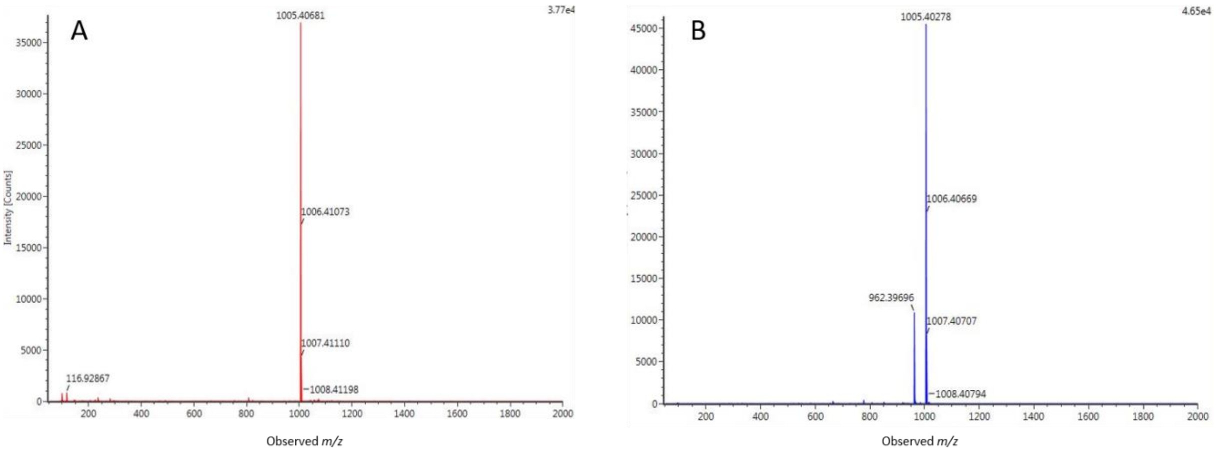

S1.4 ESI⁻-IMS-MS spectra of suomilide D. A: Low- energy collision spectrum; B: High-energy collision spectrum (20-60 eV ramp) in red.

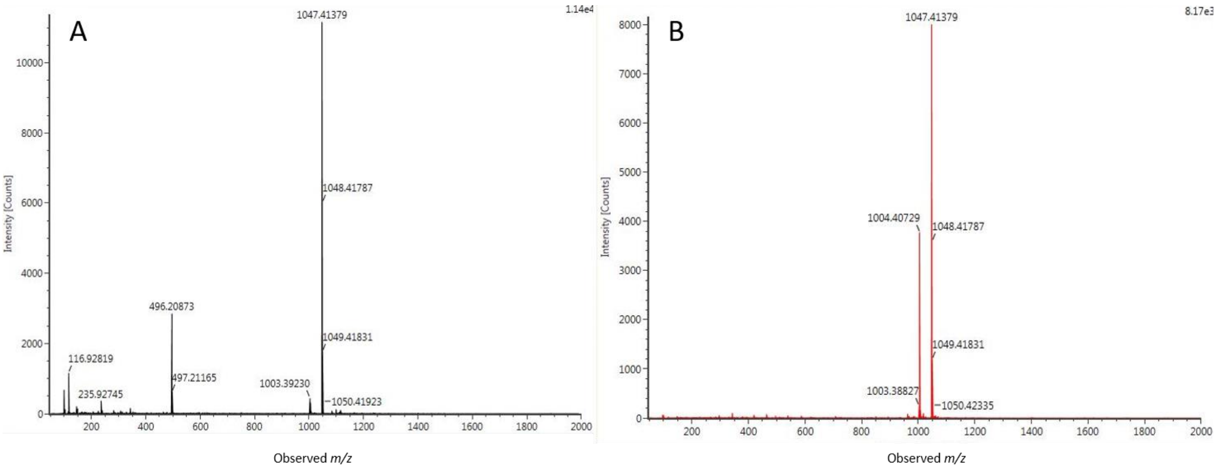

S1.5 ESI⁻-IMS-MS spectra of suomilide E. A: Low- energy collision spectrum; B: High-energy collision spectrum (20-60 eV ramp) in red.

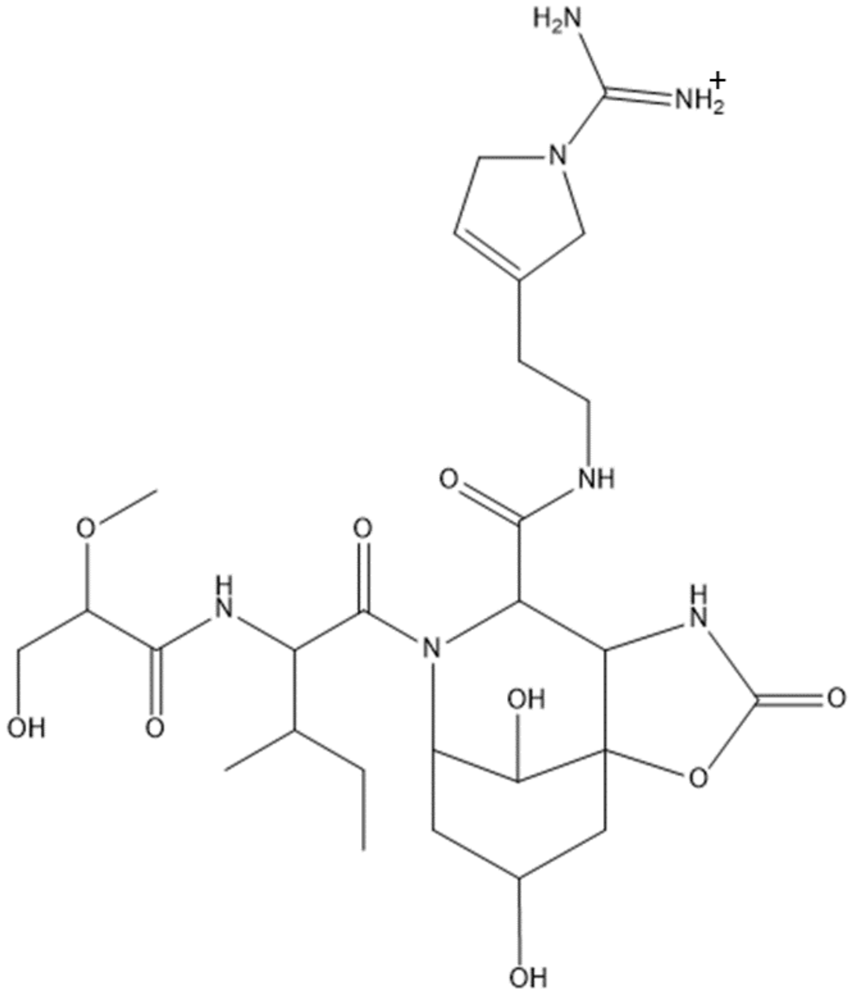

S1.6 Proposed protonated fragment for 610 (S1.1). After hydrolytic loss of sulphate and glycosyl.

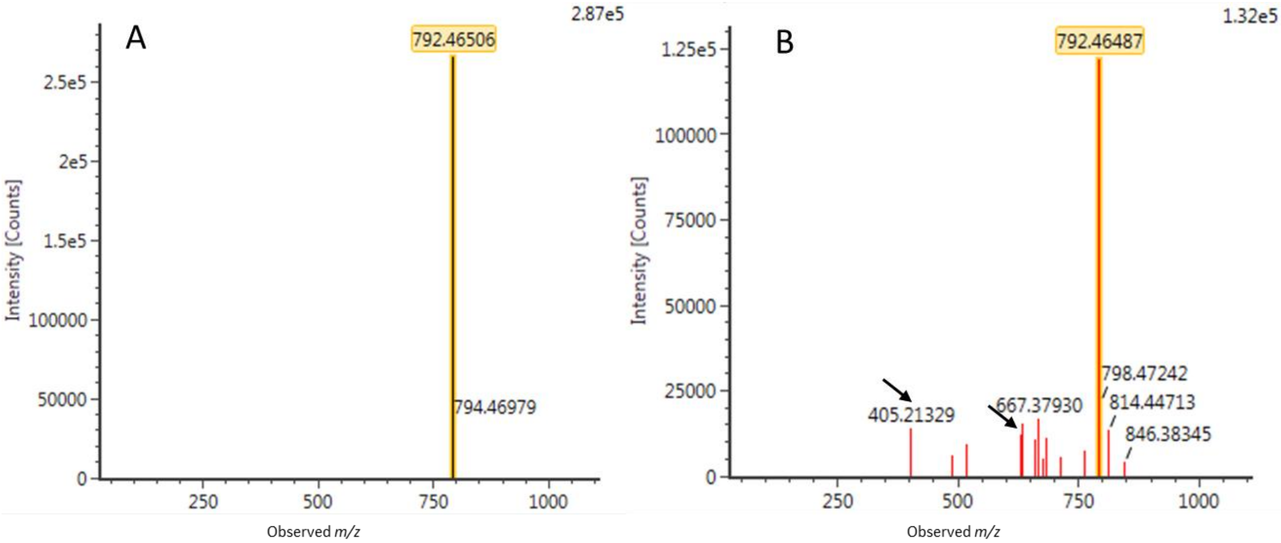

S2. ESI$^+$-IMS-MS identification of putative schizopeptin 791. A: Low-energy collision spectrum. B: High- energy collision spectrum (20-60 eV ramp), for assignment of fragments see Figure S3.

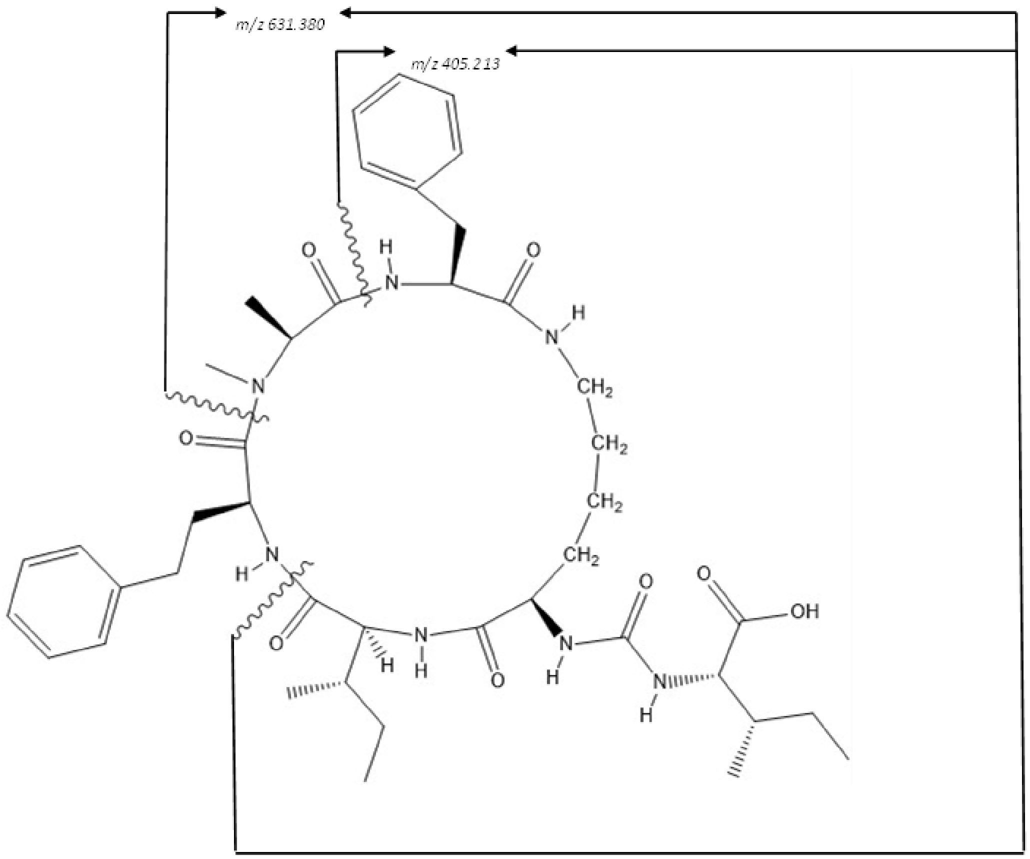

S3. Putative identification of schizopeptin 791 *via* its fragments *m/z* = 631.380 & *m/z* = 450.213 (see corresponding HR-MS data in Figure S2).

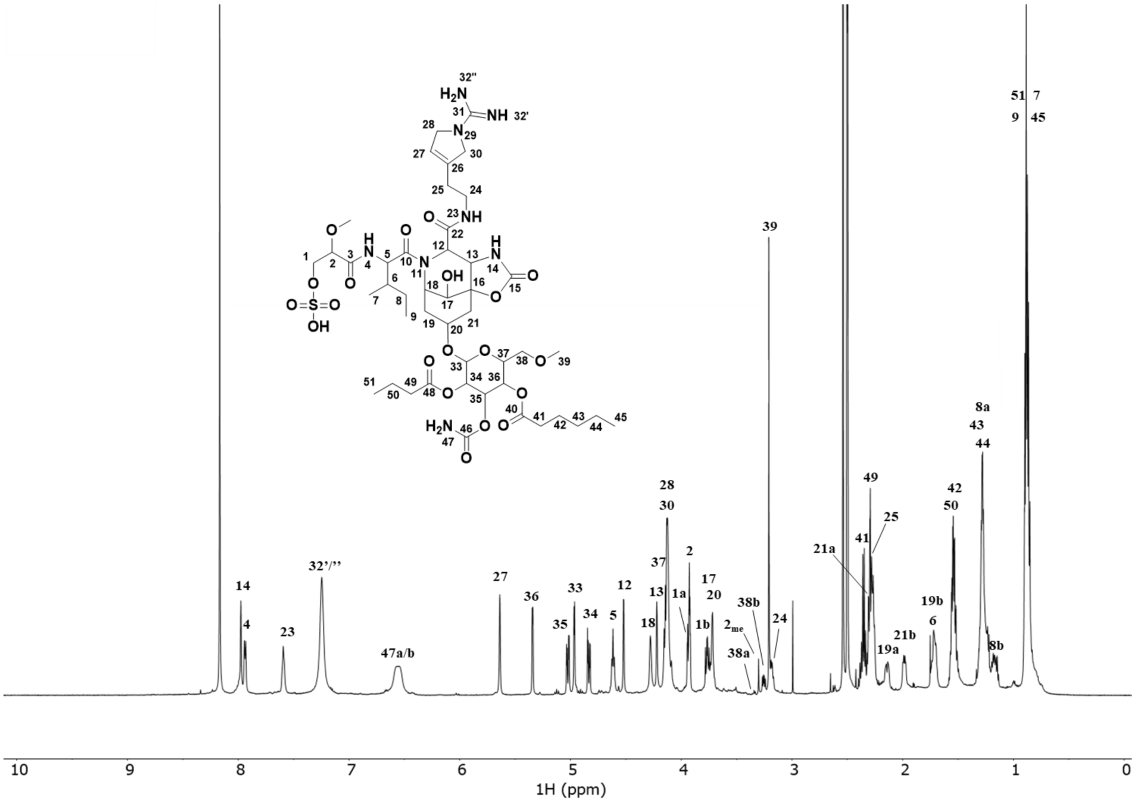

S4. ^{1}H NMR (600 MHz, DMSO-d_6) spectrum of suomilide B (**1**)

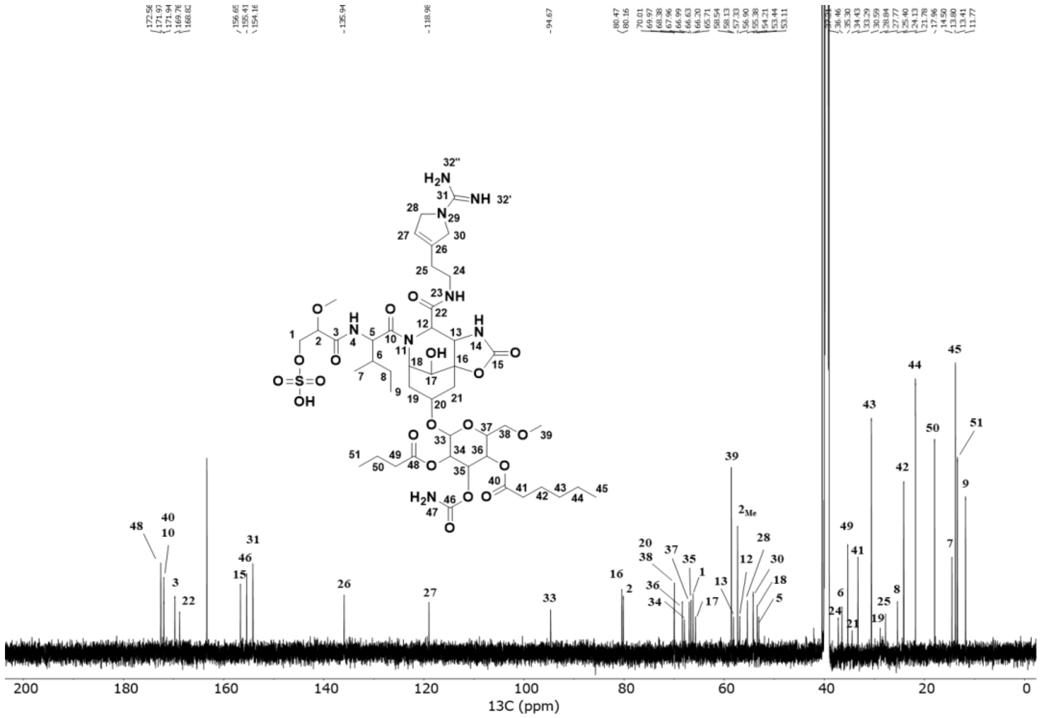

S5. ^{13}C (151 MHz, DMSO-d_6) spectrum of suomilide B (**1**)

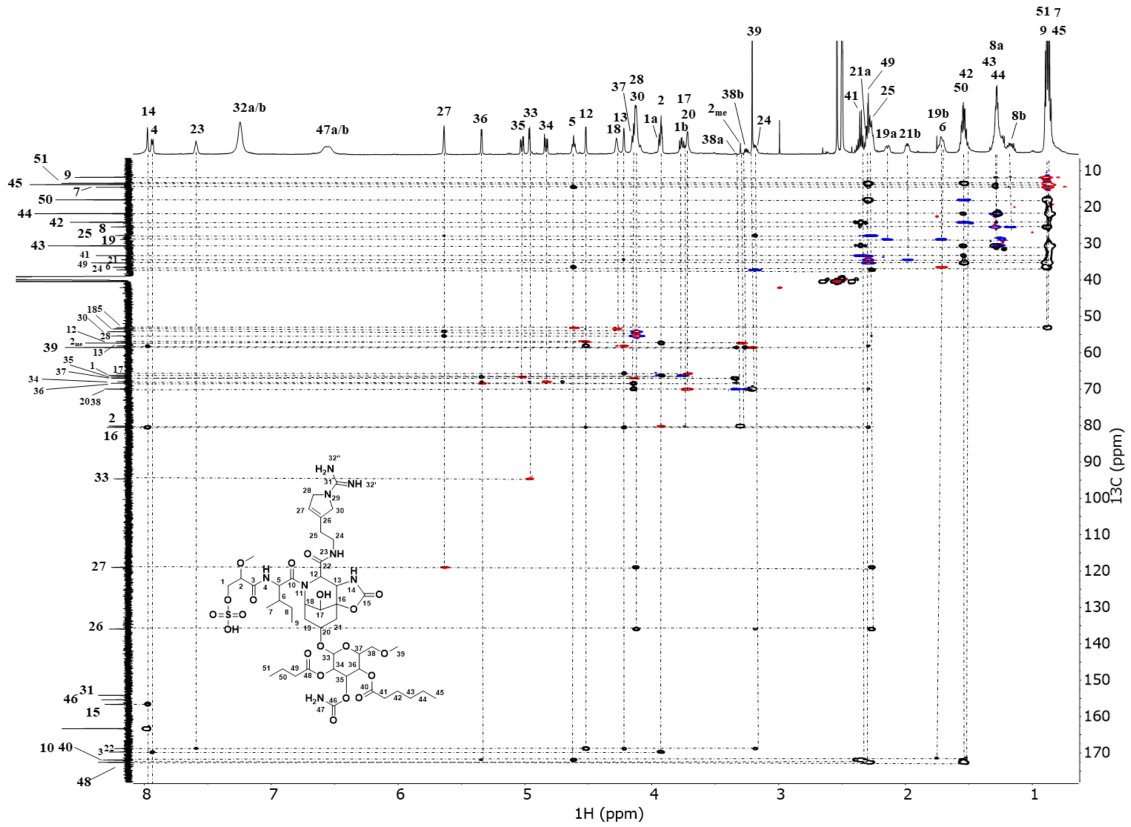

S6. HSQC + HMBC (600 MHz, DMSO-d_6) spectrum of suomilide B (**1**)

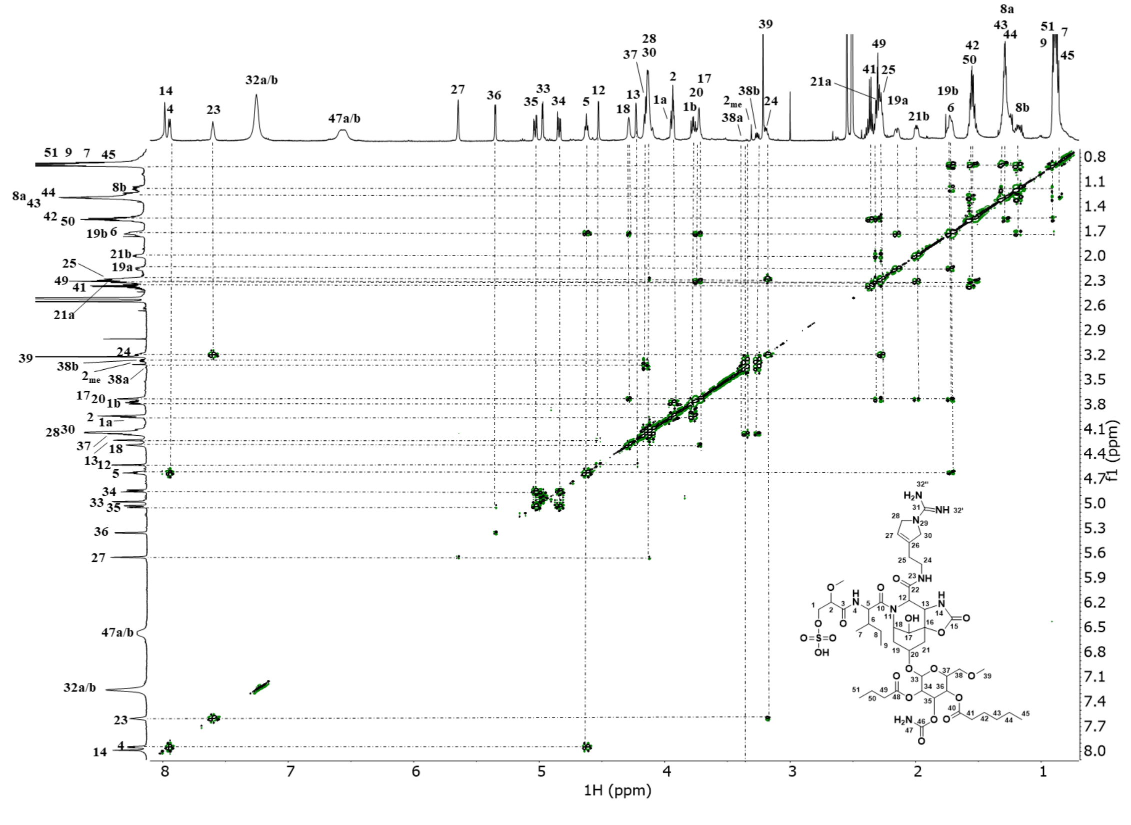

S7. COSY (600 MHz, DMSO-d_6) spectrum of suomilide B (**1**)

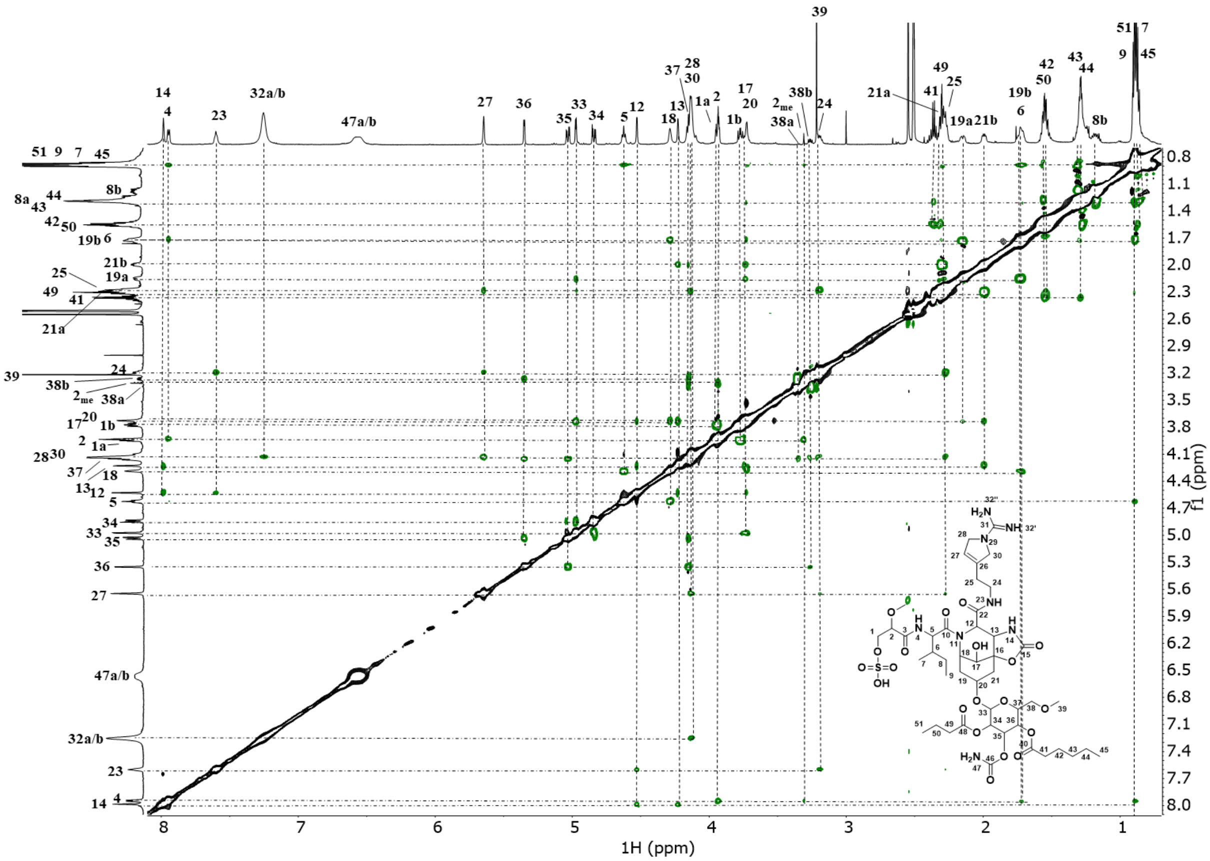

S8. ROESY (600 MHz, DMSO-d_6) spectrum of suomilide B (**1**)

S9. Comparison of 1D NMR chemical shift values between the azabicyclononane substructures of suomilide B (**1**) and suomilide (**7**) [1].

	Suomilide B (**1**)		Suomilide			
position	δ_C	δ_H	δ_C	δ_H	$\Delta\delta_C$	$\Delta\delta_H$
12	56,9	4,52	56,6	4,59	0,3	0,07
13	58,3	4,22	58,1	4,27	0,2	0,05
14		7,98		7,95	0	0,03
15	156,7		156,6		0,1	
16	80,5		80,4		0,1	
17	65,7	3,72	65,7	3,69	0	0,03
18	53,4	4,28	53,5	4,23	0,1	0,05
19a	28,8	2,14	28,7	2,14	0,1	
19b		1,72		1,68		0,04
20	70	3,72	69,8	3,62	0,2	0,1
21a	34,4	2,41 - 2,24	34,5	2,27	0,1	n.a.

21b	1,99	1,94		0,005
22	168,8	168,7	0,1	
		Avg:	0,12	0,03

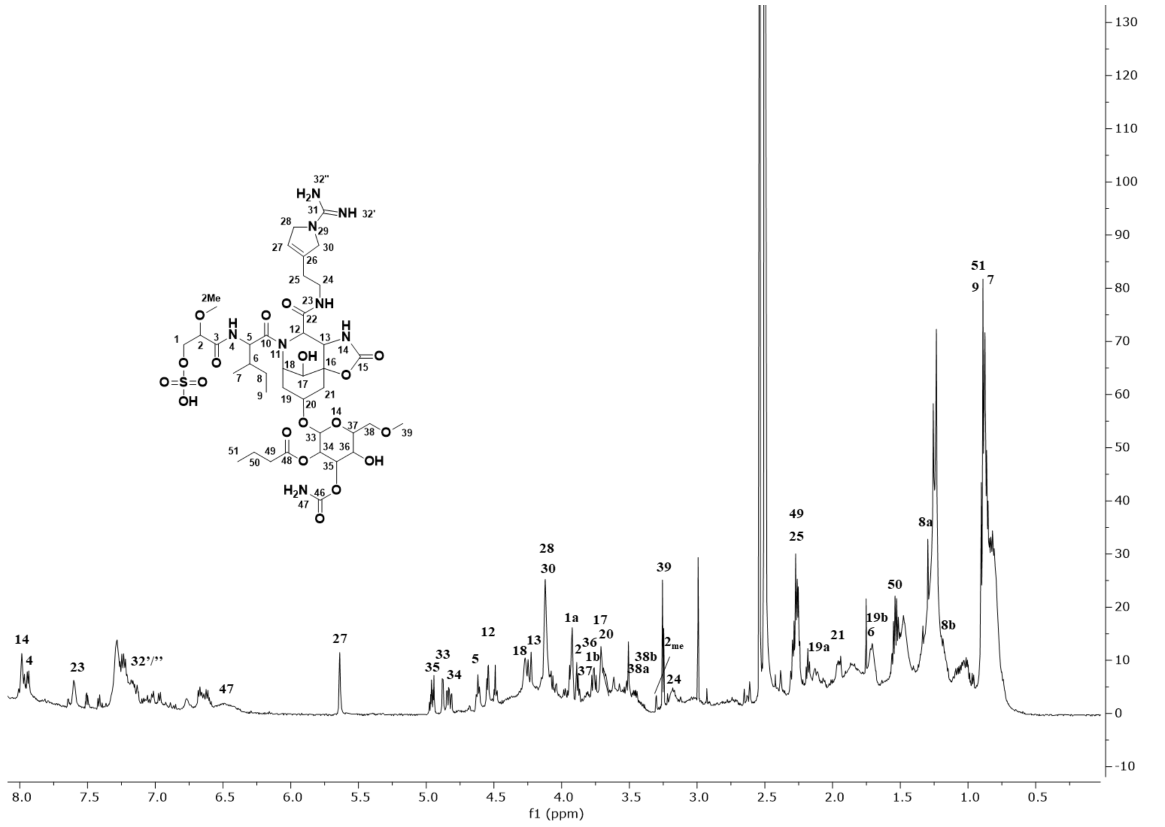

S10. ^{1}H NMR (600 MHz, DMSO-d_6) spectrum of suomilide C (**2**)

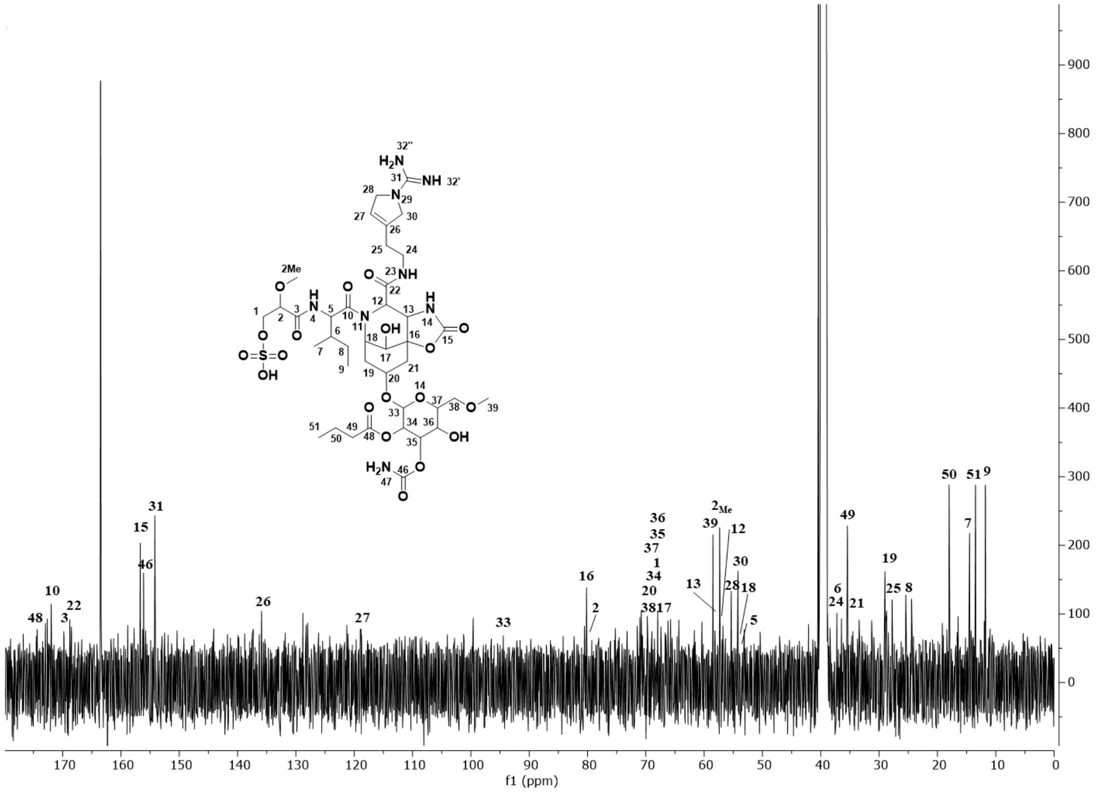

S11. ^{13}C (151 MHz, DMSO-d_6) spectrum of suomilide C (**2**)

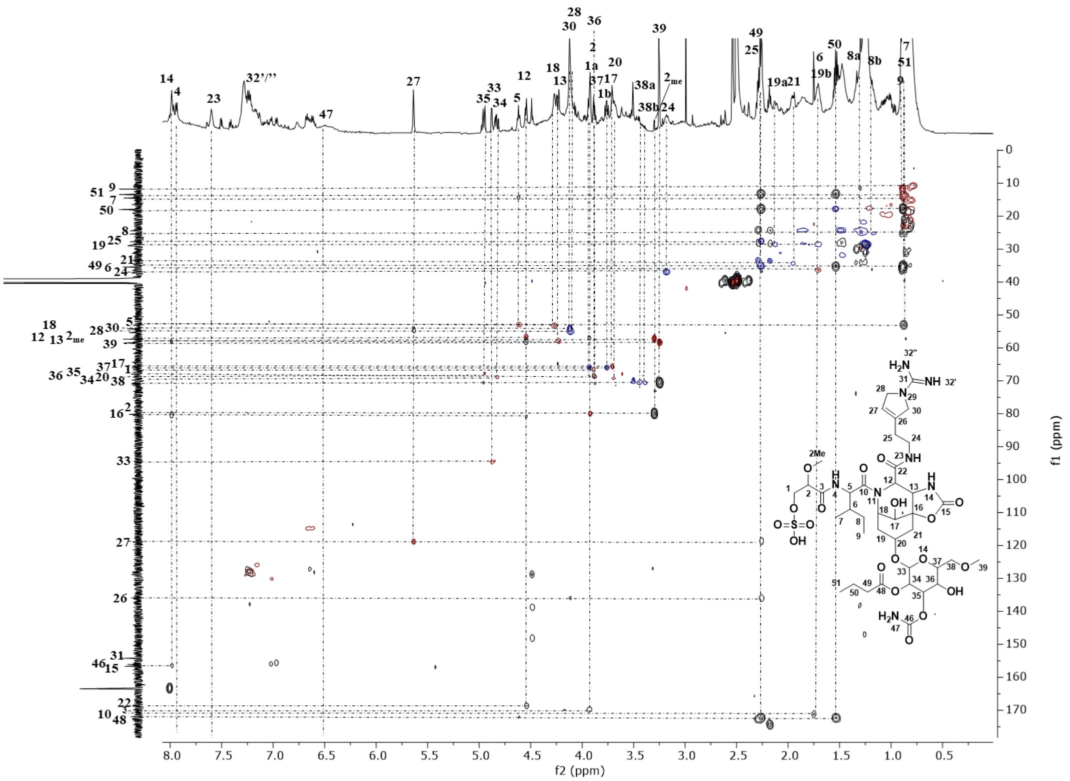

S12. HSQC + HMBC (600 MHz, DMSO-d_6) spectrum of suomilide C (**2**)

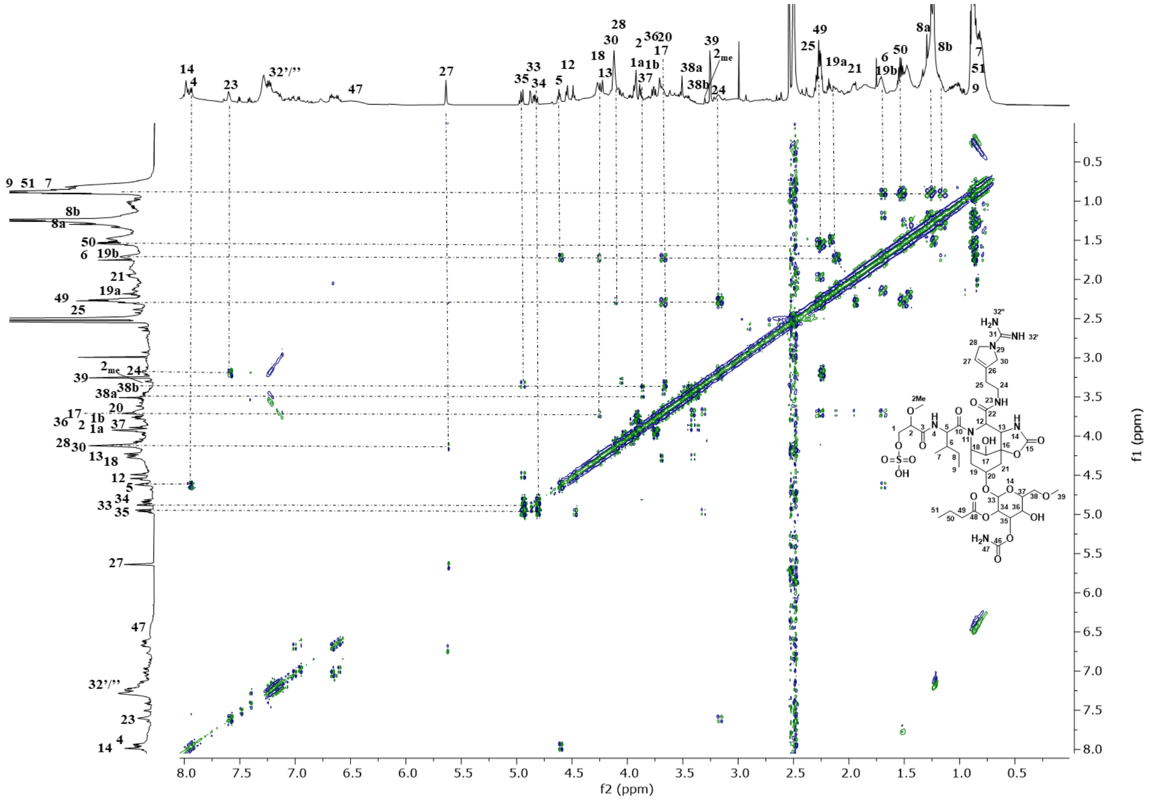

S13. COSY (600 MHz, DMSO-d_6) spectrum of suomilide C (**2**)

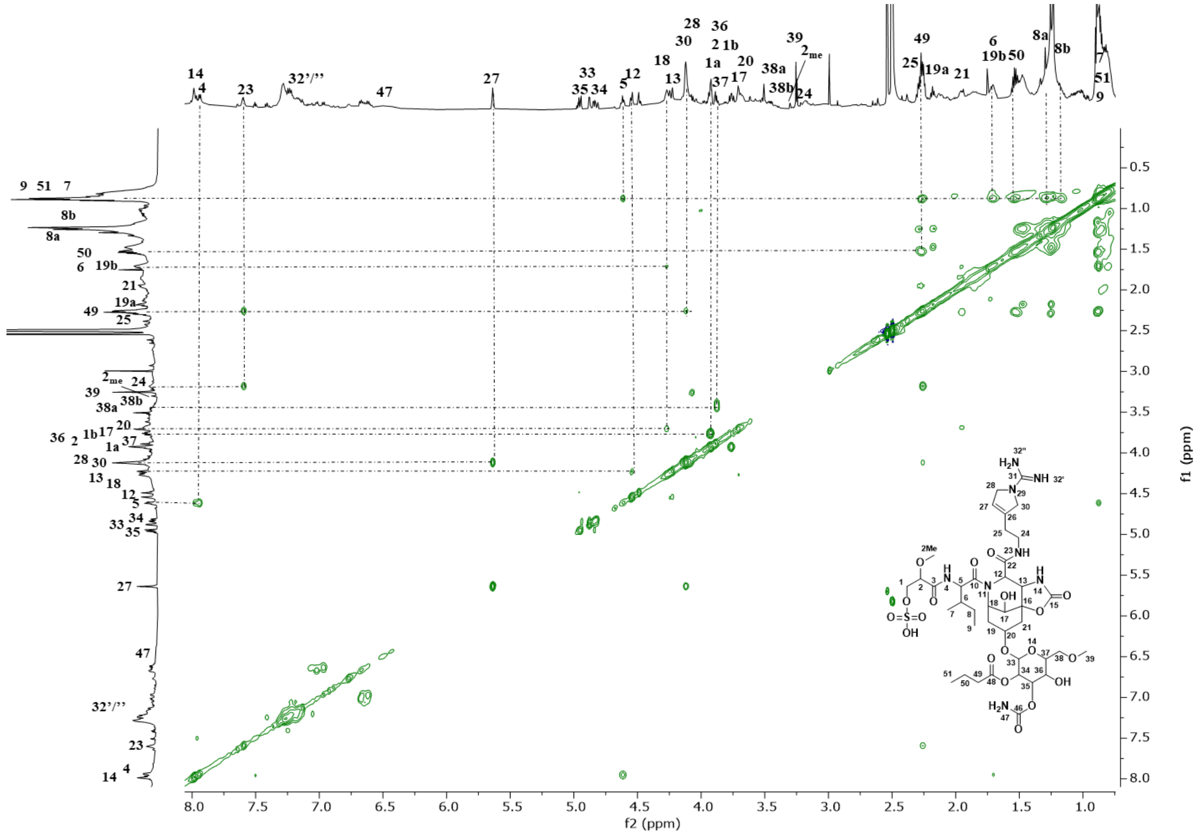

S14. TOCSY (600 MHz, DMSO-d_6) spectrum of suomilide C (**2**)

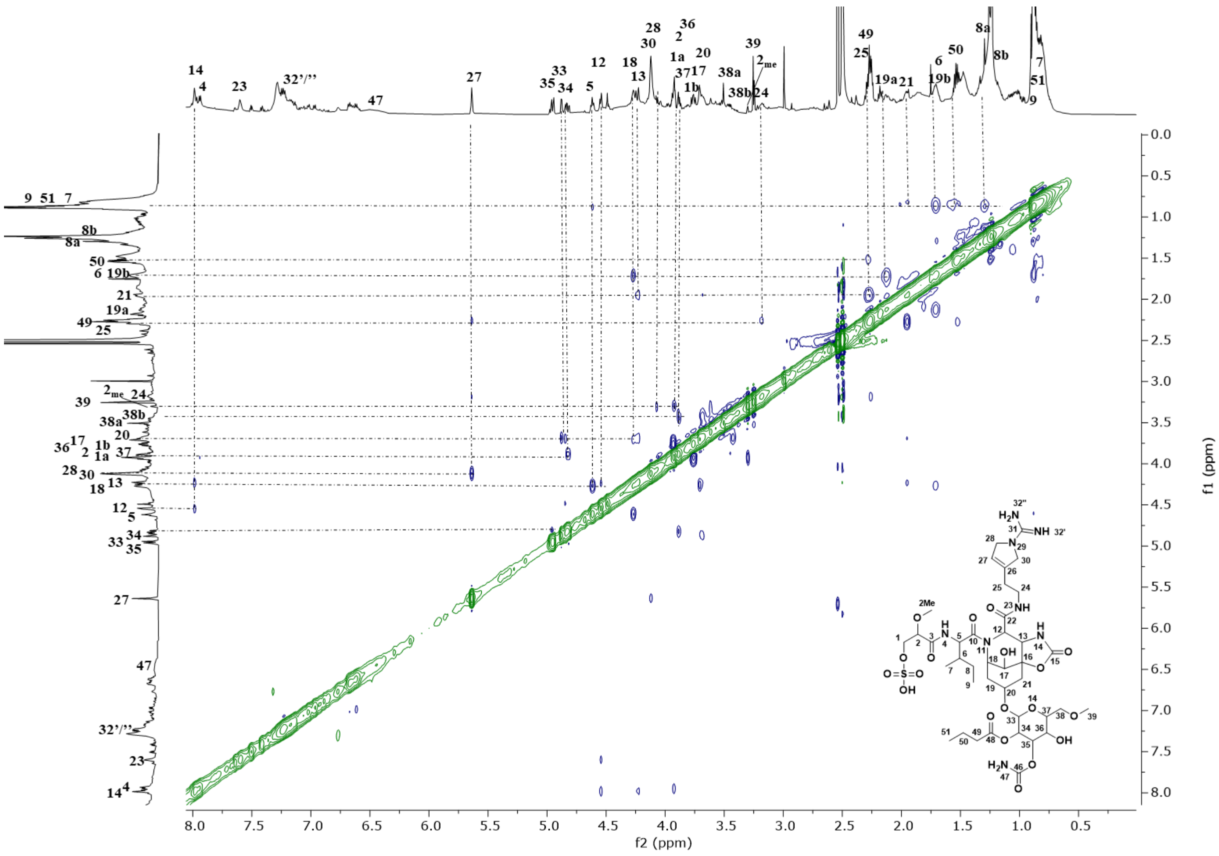

S15. ROESY (600 MHz, DMSO-d_6) spectrum of suomilide C (**2**)

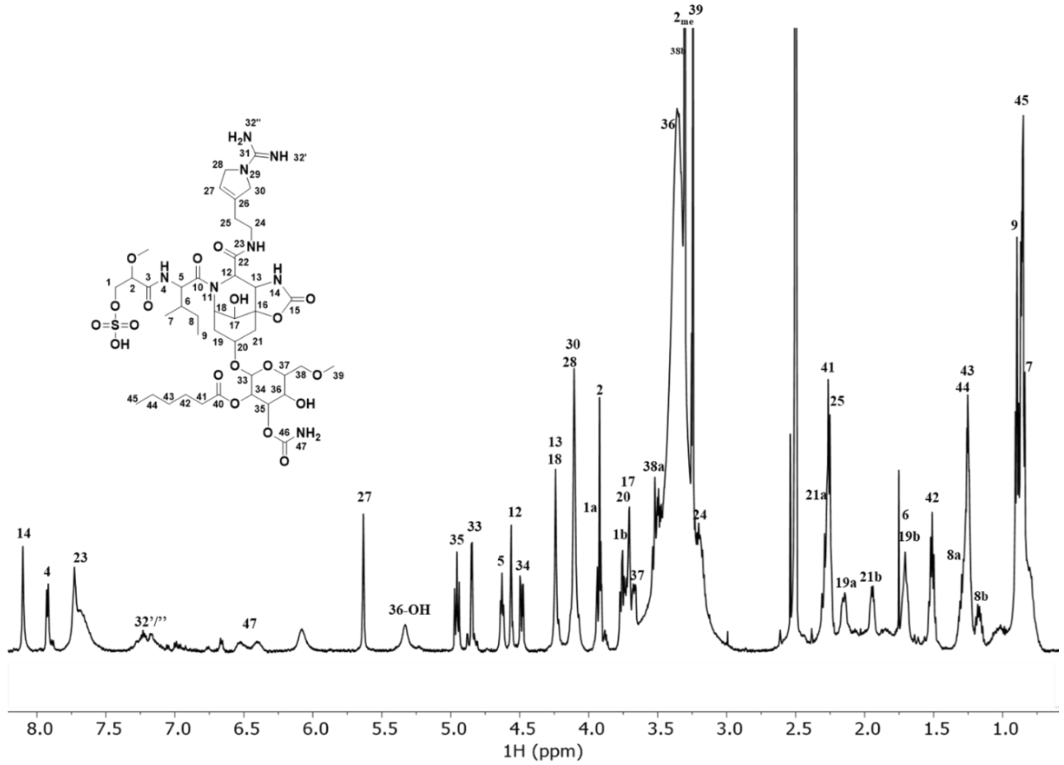

S16. ^{1}H NMR (600 MHz, DMSO-d_6) spectrum of suomilide D (**3**)

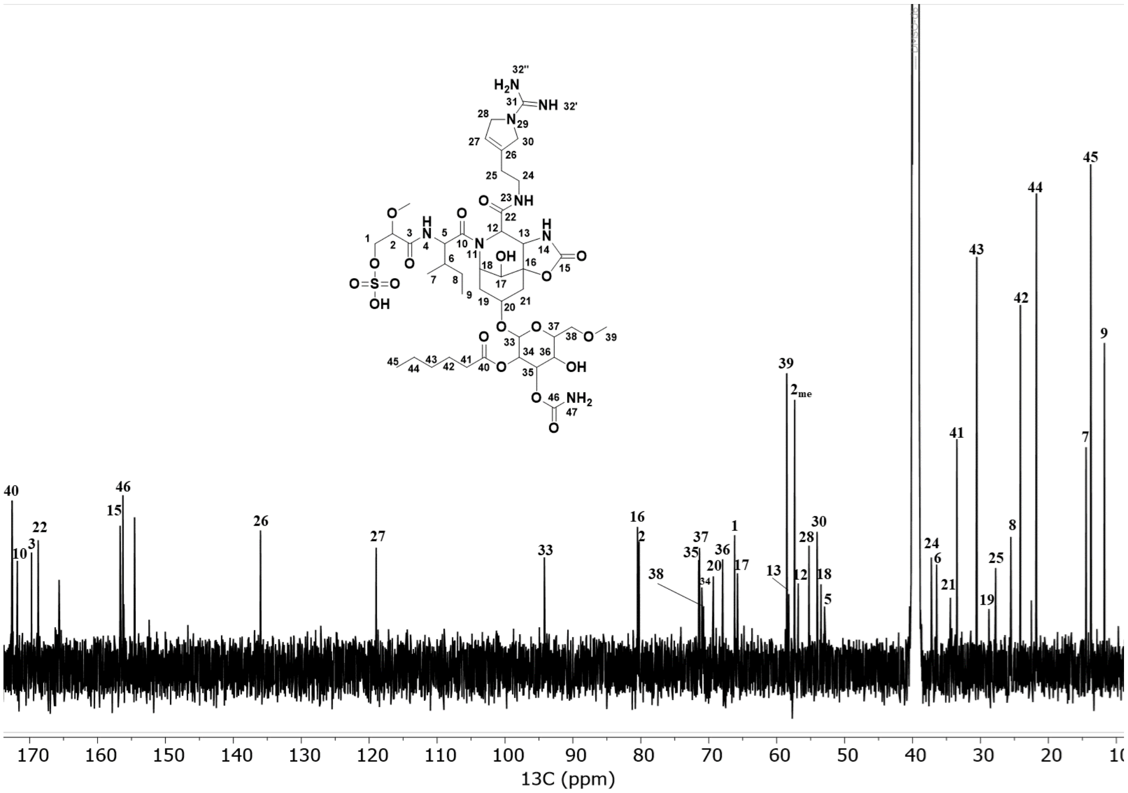

S17. ^{13}C (151 MHz, DMSO-d_6) spectrum of suomilide D (**3**)

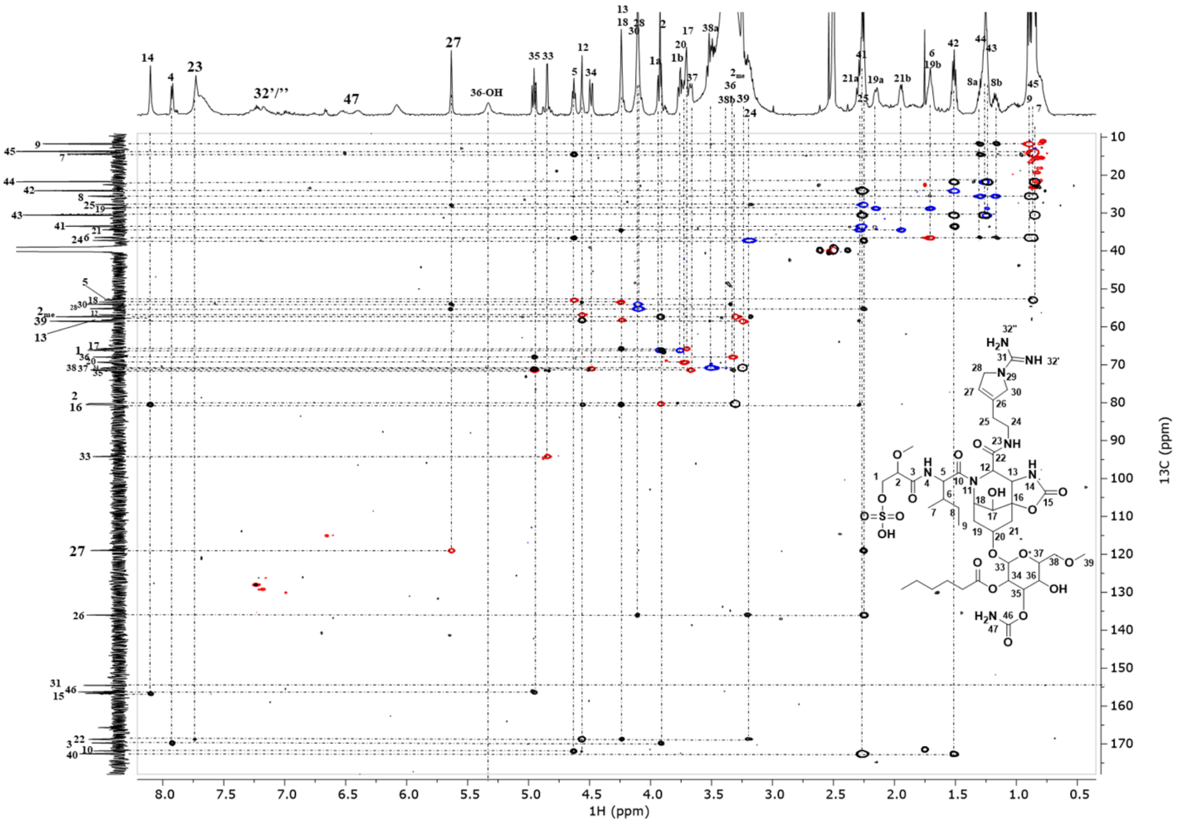

S18. HSQC + HMBC (600 MHz, DMSO-d_6) spectrum of suomilide D (**3**)

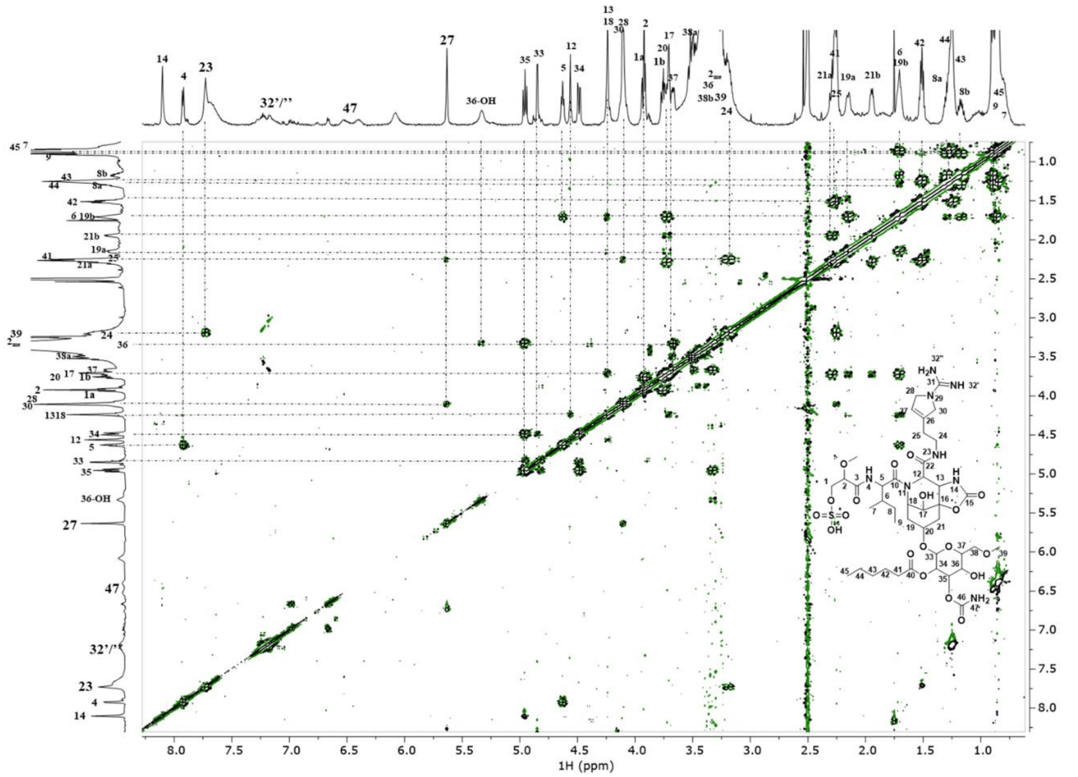

S19. COSY (600 MHz, DMSO-d_6) spectrum of suomilide D (**3**)

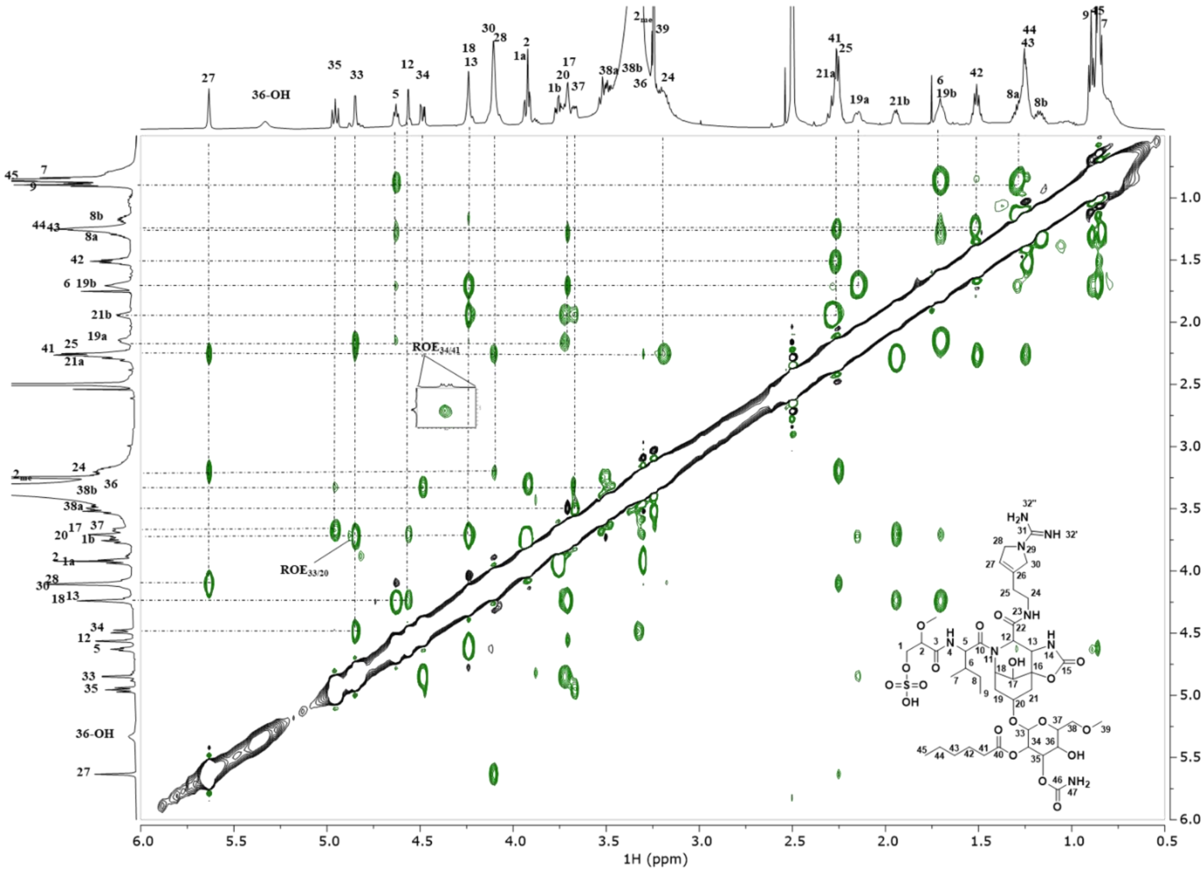

S20. ROESY (600 MHz, DMSO-d_6) spectrum of suomilide D (**3**)

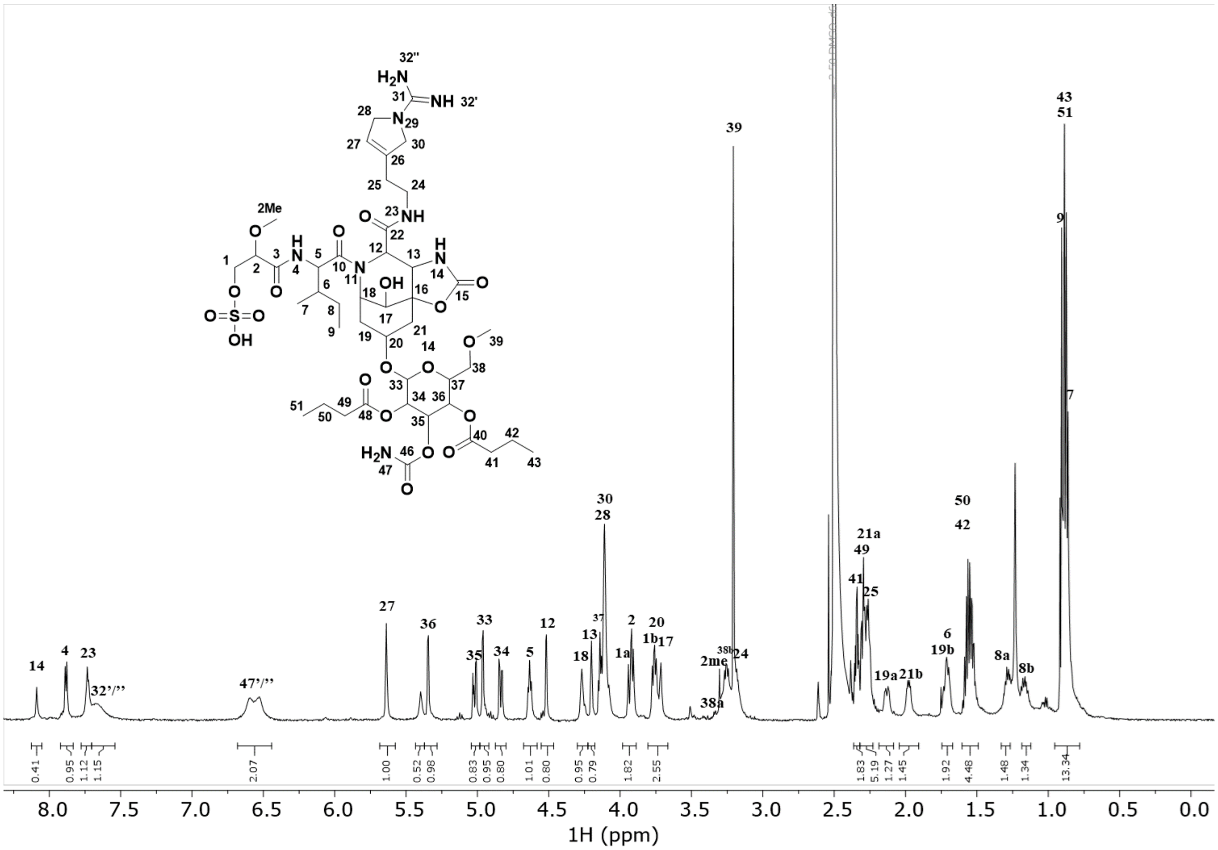

S21. ^{1}H NMR (600 MHz, DMSO-d_6) spectrum of suomilide E (**4**)

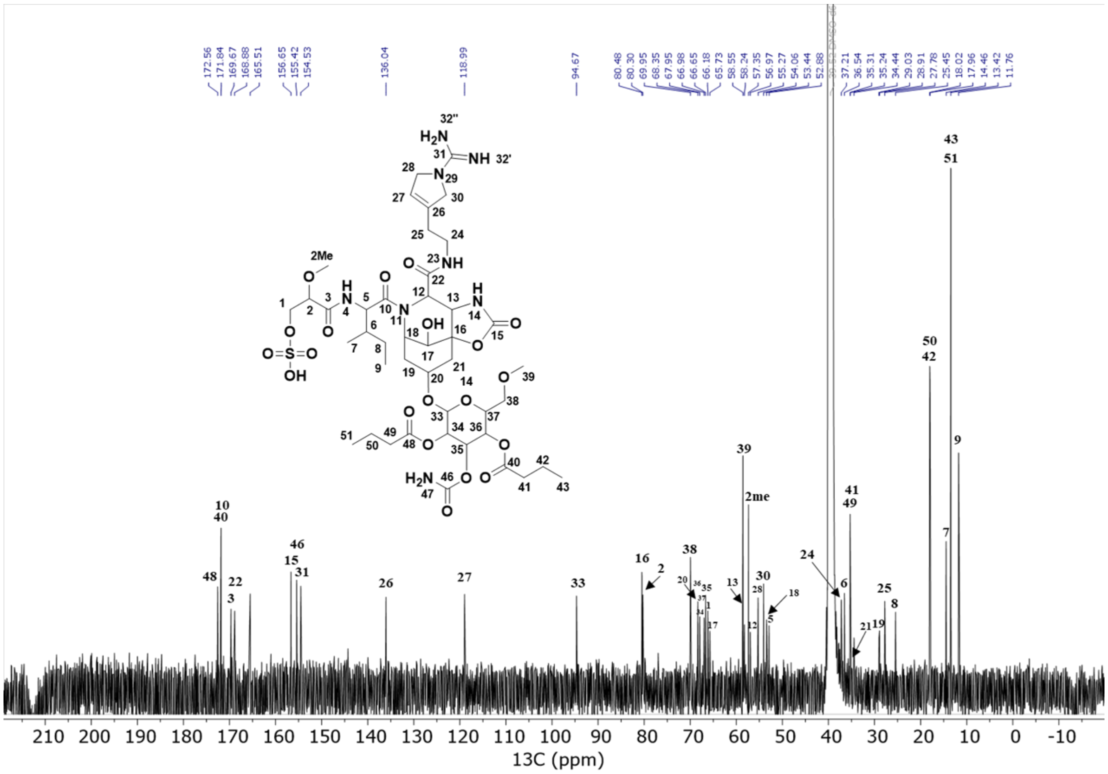

S22. ^{13}C (151 MHz, DMSO-d_6) spectrum of suomilide E (**4**)

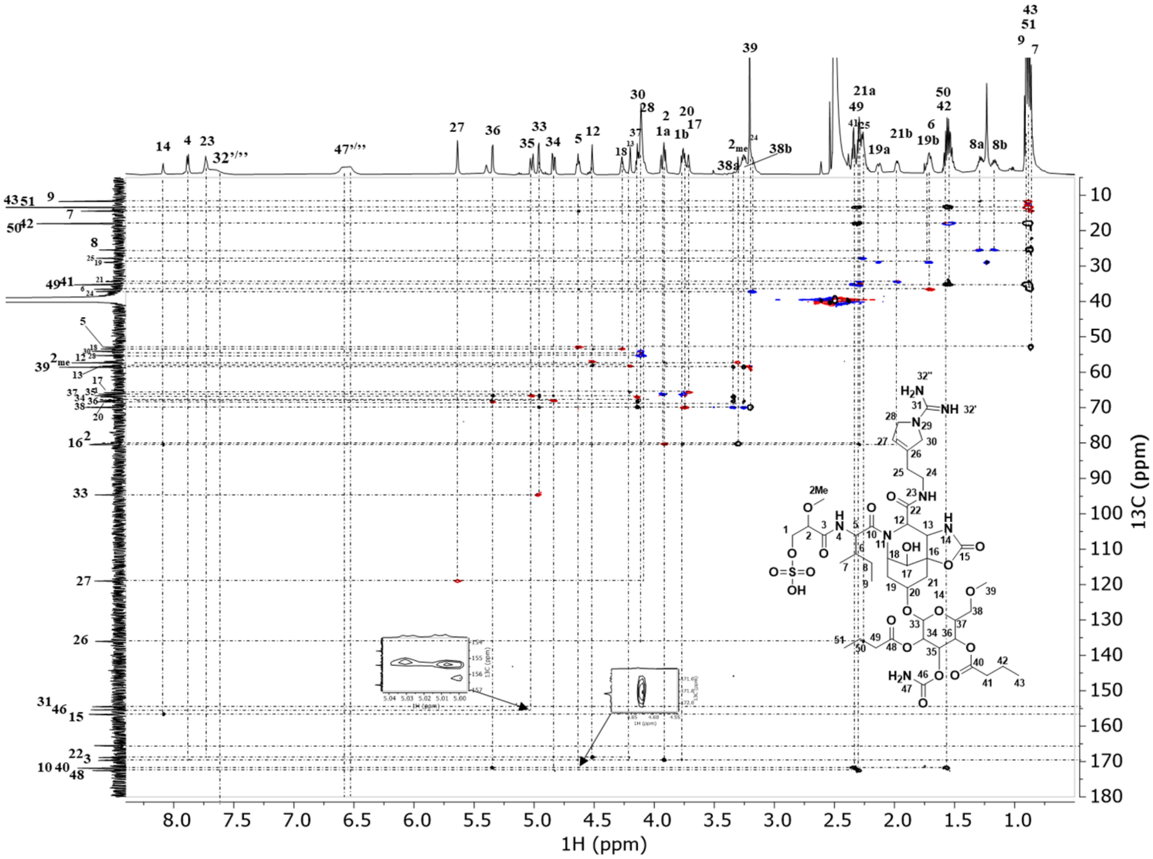

S23. HSQC + HMBC (600 MHz, DMSO-d_6) spectrum of suomilide E (**4**)

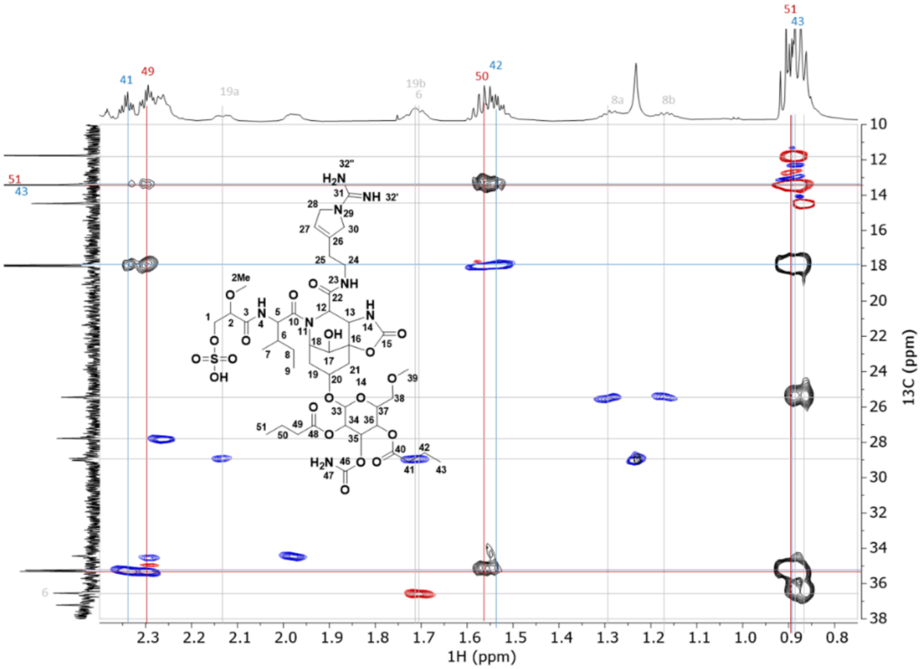

S24. HSQC + HMBC (600 MHz, DMSO-d_6) spectrum highlighting selected correlations used to assign suomilide E (**4**)

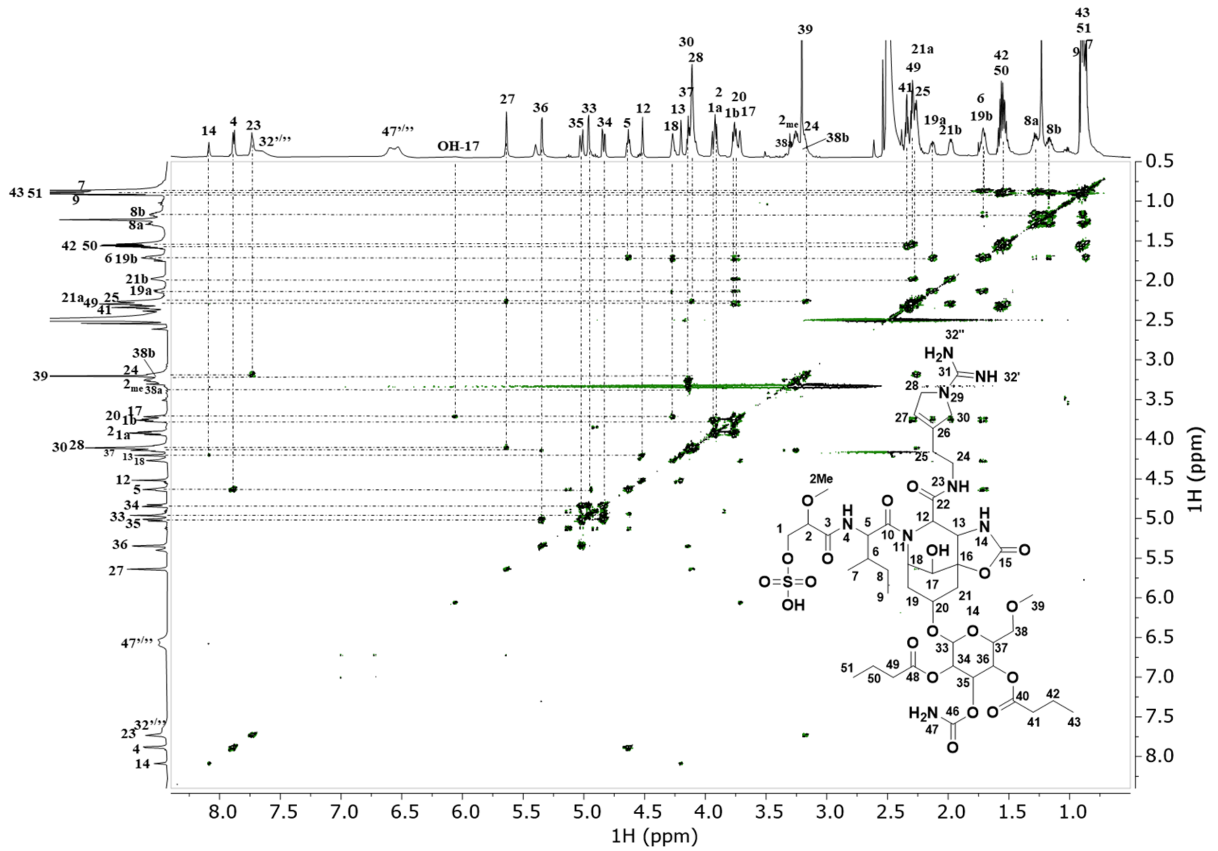

S25. COSY (600 MHz, DMSO-d_6) spectrum of suomilide E (**4**)

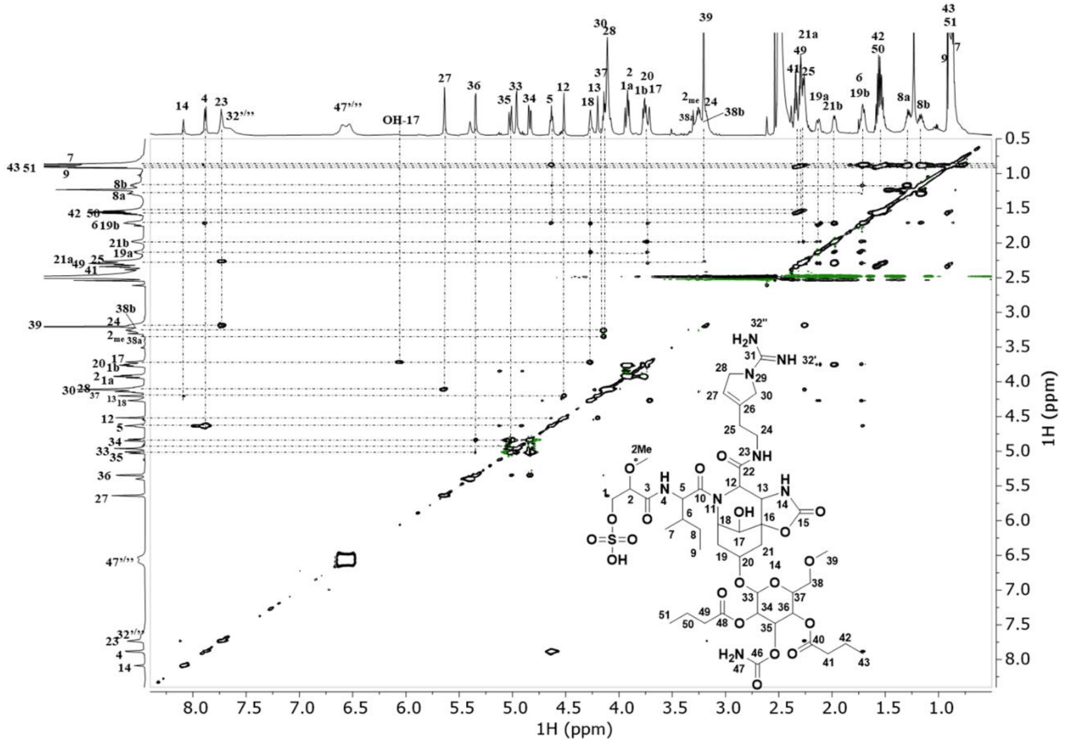

S26. TOCSY (600 MHz, DMSO-d_6) spectrum of suomilide E (**4**)

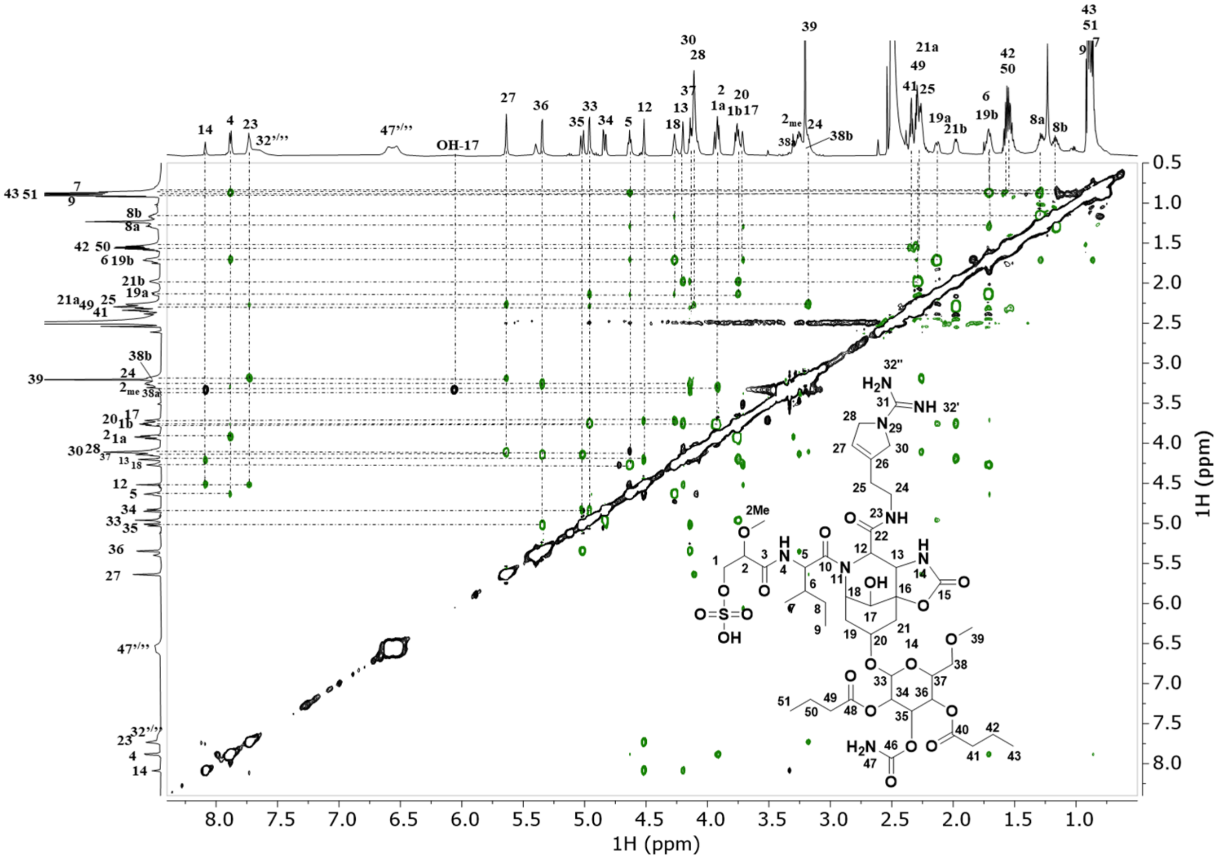

S27. ROESY (600 MHz, DMSO-d_6) spectrum of suomilide E (**4**)

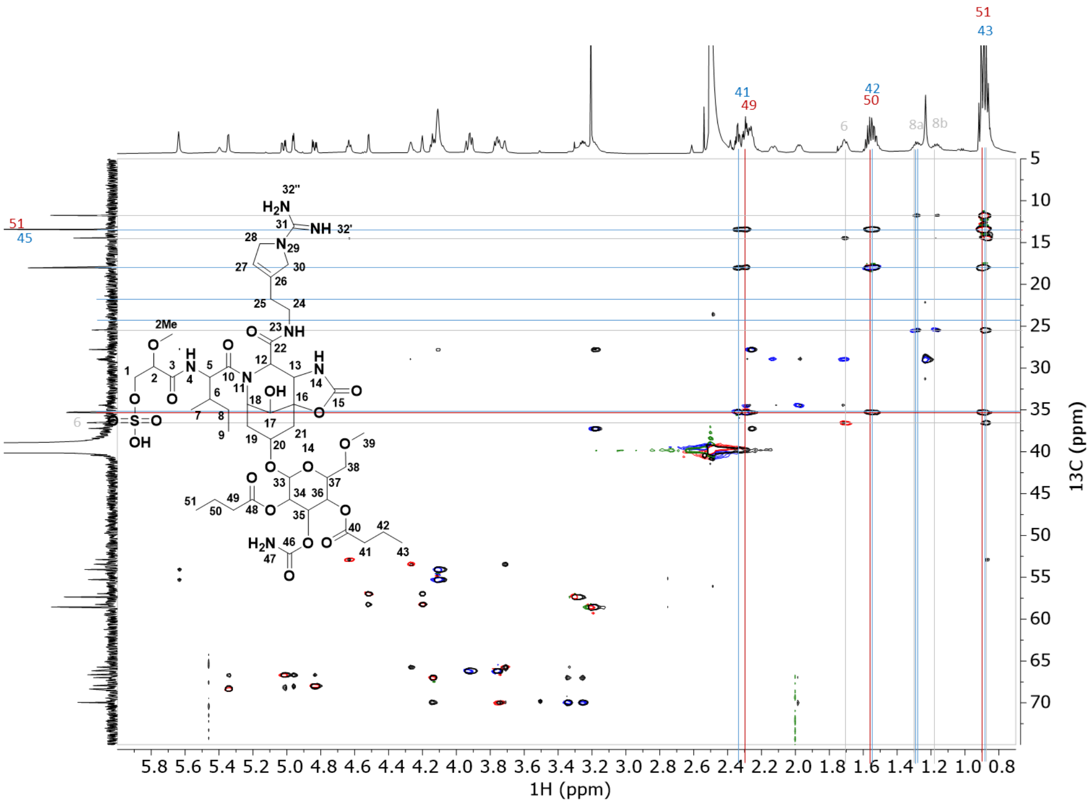

S28. HSQC + HSQCTOCSY (600 MHz, DMSO-d_6) spectrum highlighting selected correlations used to assign suomilide E (**4**)

S29. Genes of the identified bsl-cluster

Gene name	Predicted function / protein family	(Strand) ORF start:stop	BLAST match / % identity	Homology to known genes / compound /organism / notes	Proposed function	Ref.
bslA	D-Ile specific NRPS	(+) 1373:10153	1 WP_146110921 *Nostoc* sp. *'Peltigera membranacea* cyanobiont' N6 / 88%	*aerB* / aeruginosine / *M. aeruginosa*, *P. agardhii*	Incorporation of D-Ile	2, 3
bslB	Hypothetical	(+) 10244:11068	WP_162397141 *Nostoc* sp. B(2019) / 92%	*orf3* / aeruginosine / *P. agardhii*	Unknown	2
bslC	Adenylylsulfate kinase	(+) 11221:11799	WP_169266147 *Brasilonema octagenarum* / 91%	*sxtO* / saxitoxin / *Dolichospermum circinale*	Sulfatation	4

bslD	Oxidoreductase/ Ferredoxin	(+) 11821:13986	WP_094328884 *Nostoc* sp. '*Peltigera membranacea* cyanobiont' 213 / 87%	*aerC* /aeruginosine / *M. aeruginosa*, *P. agardhii*	Electron transfer	2, 3
bslE	Type 2 isopentenyl-diphosphate Delta-isomerase	(+) 13983:15038	WP_096564146 *Scytonema* sp. NIES-4073 / 95%	*aerK* / aeruginosine / *M. aeruginosa*	DMAPP synthesis	5
bslF	DMT family transporter	(+) 15071:15973	WP_162397144 *Nostoc* sp. B(2019)/ 90%		Transporter	
bslG	Dehydration and decarboxylation of prephenate, BacA	(+) 16026:16634	AVH65362 *Nostoc* sp. '*Peltigera membranacea* cyanobiont' N6 / 93%	*aerD* / aeruginosine / *M. aeruginosa*, *P. agardhii*	Dehydration and decarboxylation of prephenate	2, 3, 5
bslI	Cupin domain-containing protein, BacB	(+) 16627:17334	WP_094328882 *Nostoc* sp. '*Peltigera membranacea* cyanobiont' 213	*aerE* / aeruginosine / *M. aeruginosa*, *P. agardhii*		2, 3
bslH	SDR family oxidoreductase	(+) 17348:18142	WP_162397147 *Nostoc* sp. B(2019)/ 89%	*aerF / aeruginosine / M. aeruginosa*, *P. agardhii*	Oxidation of OH	**2, 3**
bslJ	Pro specific NRPS	(+) 18206:23224	WP_104900371 *Nostoc* sp. '*Peltigera membranacea* cyanobiont' N6 / 88%	*aerG / aeruginosine / M. aeruginosa*, *P. agardhii*	Incorporation of Abn	2, 3
bslK	SDR family oxidoreductase	(+) 23472:24248	WP_094328879 *Nostoc* sp. '*Peltigera membranacea* cyanobiont' 213 / 93%	Orf1 / aeruginosine / *M. aeruginosa*	Oxidation of OH	2
bslL	ABC transporter substrate-binding protein	(+) 24281:26077	WP_096564141 *Scytonema* sp. NIES-4073 / 88%		Transporter	
bslM	Isopenicillin N synthase family oxygenase	(+) 26228:27190	WP_104900374 *Nostoc* sp. '*Peltigera membranacea* cyanobiont' N6 / 92%	*aerH / aeruginosine /*	Unknown	2, 3

				M. aeruginosa, P. agardhii		
bslN	Isopenicillin N synthase family oxygenase	(+) 27228:28220	WP_162397152 *Nostoc* sp. B(2019)/ 85%	*aerH / aeruginosine / M. aeruginosa, P. agardhii*	Unknown	[2, 3]
bslO	Hypothetical	(+) 28286:29443	AVH65370 *Nostoc* sp. '*Peltigera membranacea* cyanobiont' N6 / 93% OYD97159 *Nostoc* sp. '*Peltigera membranacea* cyanobiont' 213 / 93%		Unknown	
bslP	D-alanyl-lipoteichoic acid acyltransferase DltB, MBOAT superfamily	(+) 29495:31012	AVH65371 *Nostoc* sp. '*Peltigera membranacea* cyanobiont' N6 / 93% OYD97158 *Nostoc* sp. '*Peltigera membranacea* cyanobiont' 213 / 93%		Acetyltransferase	
bslQ	SAM-dependent methyltransferase	(+) 31040:31762	WP_094328873 *Nostoc* sp. '*Peltigera membranacea* cyanobiont' 213 / 91%		Methyltransferase	
bslR	Carbamoyl-transferase	(+) 31810:33648	WP_094328872 *Nostoc* sp. '*Peltigera membranacea* cyanobiont' 213 / 97%	*sxtI /* saxitoxin / *Dolichospermum circinale*	Carbamoylation	[5]
sxtJ		(+) 33654:34061	WP_162397157 Nostoc sp. B(2019)/ 88%	*sxtJ /* saxitoxin / *Dolichospermum circinale*	Unknown	[5]
bslS	Hypothetical	(+) 34061:34225	2 WP_007355070 *Kamptonema* sp. PCC 6506 / 76%	*sxtK /* saxitoxin / *Dolichospermum circinale*	Unknown	[5]
bslT1	SGNH/GDSL hydrolase	(+) 34641:35552	WP_162397158 Nostoc sp. B(2019)/ 83%	*sxtL /* saxitoxin / *Dolichospermum circinale*	Hydrolase	[5]
bslT2	SGNH/GDSL hydrolase	(+) 35634:36758	WP_096564131 Scytonema sp. NIES-4073 / 91%	*sxtL /* saxitoxin / *Dolichospermum circinale*	Hydrolase	[5]
bslU1	Hypothetical	(+) 36891:37856	WP_096564130 Scytonema sp. NIES-4073 / 87%	Annotated as carbohydrate – binding protein in other	Carbohydrate binding protein ?	

				annotated genomes		
bslU2	Hypothetical	(+) 37961:38860	AVH65379 *Nostoc* sp. '*Peltigera membranacea* cyanobiont' N6 / 87% OYD97151 *Nostoc* sp. '*Peltigera membranacea* cyanobiont' 213 / 87%	Annotated as carbohydrate – binding protein in other annotated genomes	Carbohydrate binding protein ?	
bslV	Radical SAM protein	(+) 38930:39610	WP_171978128 Brasilonema / 95%		Methylation or sulphur activation	
bslW1	Isopenicillin N synthase family oxygenase	(+) 39713:40720	AVH65381 *Nostoc* sp. '*Peltigera membranacea* cyanobiont' N6 / 93% OYD97149 *Nostoc* sp. '*Peltigera membranacea* cyanobiont' 213 / 93%	*aerH / aeruginosine /* *M. aeruginosa, P. agardhii*	Unknown	[2, 3]
bslW2	Isopenicillin N synthase family oxygenase	(+) 41045:42028	OYD97148 *Nostoc* sp. '*Peltigera membranacea* cyanobiont' 213 / 93%	*aerH / aeruginosine / M. aeruginosa, P. agardhii*	Unknown	[2, 3]
bslY	Aldo/keto reductase	(-) 42143:42976	AVH65383 *Nostoc* sp. '*Peltigera membranacea* cyanobiont' N6 / 94% OYD97147 *Nostoc* sp. '*Peltigera membranacea* cyanobiont' 213 / 94%		Hydration of DB/ reduction of OH	
bslX	glycosyltransferase family 4 protein	(+) 43181:44470	AVH65384 *Nostoc* sp. '*Peltigera membranacea* cyanobiont' N6 / 94%	*aerI / aeruginosine / P. agardhii*	Glycosylation	[2]
bslZ1	MFS transporter	(+) 44638:45984	WP_096570964 *Scytonema* sp. NIES-4073 / 89%		Transporter	
bslZ2	ABC transporter, similar to anabaenopeptin transporters	(+) 46051:48060	WP_162397169 Nostoc sp. B(2019)/ 83%	*aerN / aeruginosin / M. aeruginosa / P. agardhii*	Transporter	[3]

S30. Gene expression study of BGC in *Nostoc* sp. KVJ20 subjected to different growth conditions:

Material and methods:

Cultivation and experimental set-up. Starter cultures of *Nostoc* sp. KVJ20 were cultivated for 2 weeks in liquid BG11 medium [6] under standard conditions at 23°C with constant light 30 µmol $m^{-2}s^{-1}$ (36W/77 Osram Fluora) and moderate linear shaking at 30 rpm. The bacterial cells were collected by centrifugation (Eppendorf Centrifuge 5804 R) at 5000 rpm for 5 minutes, and then homogenized using a 0.4 mm syringe (BDMicrolance™). The suspension was divided equally into six 50 ml falcon tubes and conditioned to the next step cultivation media by washing in the respective medium two times. The cell suspension was then distributed equally as 5 ml aliquots into 45 ml respective growth medium and left for cultivation for another week. The growth media were liquid BG11, BG11 without phosphate, BG11 without iron, BG11o (without nitrate), BG11o without phosphate and BG11o without iron. For the colony cultivation on solid media and allelopathy the procedure was as described in Liaimer *et al*., 2016 [7]. For the allelopathy experiments we used *Nostoc* sp. KVJ2 and *Nostoc* sp. KVJ10 from the same study. All cultivations were done in biological triplicates.

RNA extraction and gene expression analyses. Cells were collected by centrifugation or by scraping from the agar surfaces and preserved in 300 µL of RNA*later*™ Stabilizer Solution (ThermoFisher), and stored at - 20°C. The RNA extraction was conducted according to hot Trizol protocol described in Pinto et al., 2009 [8]. cDNAs were synthetized using SuperScript™ II Reverse Transcriptase (Invitrogen) according to the manufacturer's instructions. The RT-PCR were performed with SsoFast™ EvaGreen® Supermix (ThermoFischer) on CFX96™ Real-Time PCR Detection System (Bio-Rad). The primers for 19 BGCs identified by AntiSMASH and additional BLAST are listed in the table S36.1 We targeted the longest open reading frame in each BGC. Additionally we used primers for *rnpB* as internal housekeeping reference for relative expression calculations, *nifH* as reference for diazotrophic growth, *avaK* as akinete formation marker and *gvpC* as hormogonia differentiation marker. The same threshold line was used on all runs, and relative expression was calculated by the formula: RE (relative expression) = $2^{-(cQgene – cQrnpB)}$, with standard deviation (STDV), and fold changes in expression by the formula: FC (fold changes) = $2^{-(\Delta cQexp - \Delta cQref)}$. The FC values represent means of 3 biological replicates, with 2 technical replicates for each sample. Fold changes were calculated for cultures which differed only in one growth condition variable.

S31.1. Primers designed for Nostoc sp. KVJ20. N stands fro NRPS gene cluster, NP for hybrid NRPS/PKS, P for PKS gene cluster and RiPP – for clusters coding ribosomally synthesised post-translationally modified products.

**Primer**	**Cluster type**	**Product/function**	**NCBI accession and coordinates**	**Primer 1 5' to 3'**	**Primer 2 5' to 3'**
**20-RnpB**	Housekeeping, reference gene	Ribonuclease III	LSSA01000318.1: 20771-20958	ATCCTAGCTTGGTTCCGTGC	GCTCTTGGCTAGATCCCCAC
**20-NifH**	Nitrogen fixation	Nitrogenase	LSSA01000069.1: 8617-8801	GAGCGATGTTGTTAGCAGCG	CCAGAACCCGGTGTAGGTTG
**20-GvpC**	Motility, buoyancy	Gas vesicle	LSSA01000004.1: 11984-12207	CAAGAATACCGCCAACAGCG	ATCGTATTGGCGGAAGGACG
**20-AvaK**	Akinete marker gene	Membrane bound barrel	LSSA01000133.1: 14032-14209	GGCGTCGTTTACTGGCAATC	ACGGGTGCGTGGTGTATAT

**20-N1 (Bsl)**	NRPS	Suomilide	LSSA01000176.1: 6892-9906	TCCAGCTTGATTGGCTCTGG	ACGCCCAATGGCAAAGTAGA
**20-N2 (Apt)**	NRPS	Anabaenopeptin	LSSA01000425.1: 19463-22459	ATTGGGGTGGATTGCAGAGG	GCGATGAATTTTGGCGGTGT
**20-N3**	NRPS	Unknown	LSSA01000197.1: 146-6064	CAGCAAACTGTCGTCACAGC	GGGTTAGGGGTAGGGTGTCT
**20-N4 (Ncp)**	NRPS	Nostocyclopeptide	LSSA01000164.1: 29725-32772	GCAACTAGAGCAGACAGCCA	TCTTCAATAGGAGTGCGCGG
**20-NP1**	NRPS/PKS	unknown	LSSA01000013.1: 23704-28650	AAAGCAAGTTTGCCGCAGAG	TTCATGTTCCCCGGACAAGG
**20-NP2 (Sid)**	NRPS/PKS	Possible sideophore	LSSA01000336.1: 21785-28384	AACCGCTGGGTGTTGGTTTA	TAATCGCCCAGAATCGACCG
**20-P1 (Hgl)**	PKS	Heterocyst glycolipids	LSSA01000165.1: 23368-28743	TGTCCAAATTGCTGCCTTGC	CAGCCATTGCTTGACCCCTA
**20-P2**	PKS	Unknown	LSSA01000184.1: 29769-32819	GGCGGATTTATCCCGGATGT	TTCTCGCAGTCAGTGGAACC
**20-RiPP1**	RiPP	Unknown	LSSA01000401.1: 45825-47084	CAAGCCAACTGGACTGGCTA	TCAGAACGCTGTACCACCAC
**20-RiPP2**	RiPP	Unknown	LSSA01000352.1: 15209-16471	AGTGGTGCATCCCTAAAGGC	GTTGACCAAGAGCGGGATCA
**20-RiPP3**	RiPP	Unknown	LSSA01000102.1: 5744-8062	CCTGAGCAAAGGCTGGTACA	CGGGTGTTGTGCTGACAATG
**20-RiPP4**	RiPP	Unknown	LSSA01000383.1: 1971-5093	TGGATGAGGCAACCAGTCAC	CGTGAGTACCGCTTTCGACT
**20-RiPP5**	RiPP	Unknown	LSSA01000098.1: 8076-10244	CGGGGATTGCTGAGTCAAGT	TTGCAATGCTTGCAGACCAC
**20-RiPP6**	RiPP	Unknown	LSSA01000337.1: 181966-183417	CTTAGGCGCACCACAGGTTA	TGGTCACAATGGCTCTTGCT
**20-RiPP7**	RiPP	Unknown	LSSA01000346.1: 285-2513	TAGGTGCATGACGATGACGG	GTTTTGGAGGCTTTCTGCGG
**20-RiPP8 (Mvd)**	RiPP	Microviridin	LSSA01000045.1: 26899-27903	CCCAAGAATGTGAGCAGGGT	GCAATTCAAGGGCTTTGGGG
**20-RiPP9 (Mvd)**	RiPP	Microviridin	LSSA01000164.1: 41782-42756	GACCAACTTTGCGTGCAACT	TCCTCAGTTTGGTATCGGCG
**20-RiPP10 (Lan)**	RiPP,	Unknown lantibiotic	LSSA01000386.1: 2980-6204	ATCCAGTTGATGCGGCAGAA	GTCGGAGTCAGACTGGAACG
**20-RiPP11 (Lan)**	RiPP,	Unknown lantibiotic	LSSA01000045.1: 18651-21953	CGAATTCGGCTGCTCTTTCG	CCATCTGCACCATTGAACGC

Results and Discussion. The microscopy observations on the morphology of cyanobacterial cultures cultivated under nitrogen supplementation and without nitrate gave expected results. Filaments grown in $BG11_0$ differentiated nitrogen fixing cells heterocysts. Likewise, all cultures subjected to phosphate limitation developed pronounced akinetes, resting cells, as previously reported for *N. punctiforme* [9]. Iron limitation caused heterocyst differentiation in nitrogen replete cultures, a phenomenon also described earlier by Lopez-Gomollon et al., 2007 [10], while under nitrogen depletion

filaments started compacting into so called aseriate packages, believed to be a characteristic of a stationary growth stage in *Nostoc* [11]. Thus, we confirmed that the cultures had diverged in their growth in response to various limitations.

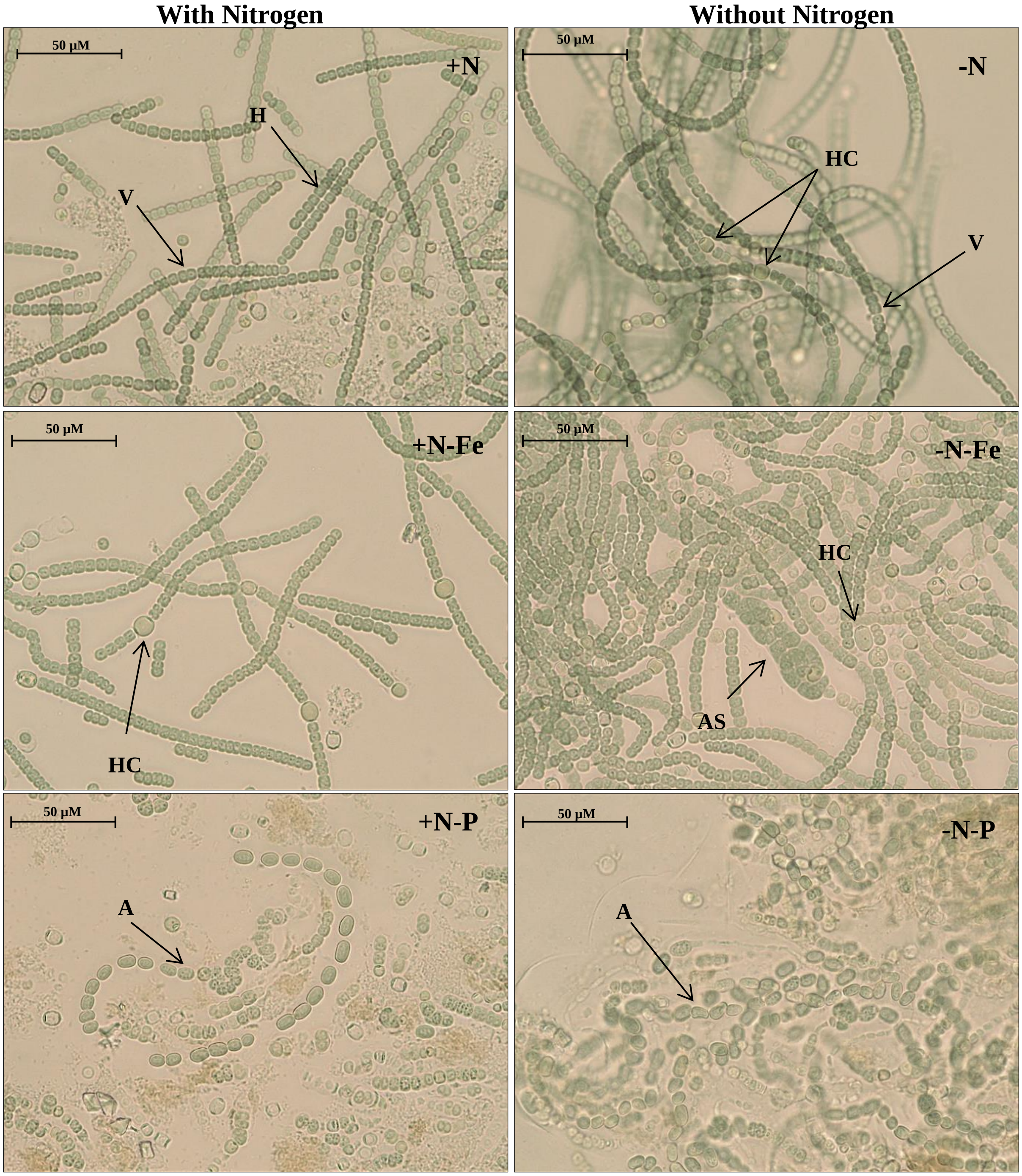

Figure S31.2. Morphological appearance of the *Nostoc* sp. KVJ20 subjected to different cultivation conditions. A-akinetes (spore-like cells), AS- aseriate packages, H-hormogonia, HC- nitrogen fixing heterocyst's, V- vegetative cells.

The expression patterns of reference genes (table S36.3) have been aligned to the observations made by microscopy. Nitrogenase was higher expressed in diazotrophically grown cultures and nitrogen supplemented cultures without iron source, which is in line with the micrographic images. Gene for gas vesicle protein (*gvpC*), the hormoginia marker, was down-regulated in diazotrophically grown cultures which is supported by the absence of motile hormogonia filaments in those. *avaK*, indicative of akinete formation, was down-regulated at the time of sampling in both phosphate deplete cultures, despite presence of akinetes. This may indicate that the gene was either expressed at the earlier stages in the process of akinete differentiation, or the gene is not involved in the process in this particular strain

In general, we noted that there was no substantial difference in overall gene expression levels in *Nostoc* colonies grown on solid medium, regardless of presence or absence of nitrogen source. This suggests that the majority of the cells grown on nitrogen supplemented agar still grow under limitation of nitrogen and possibly other nutrients.

Table S31.3. The fold changes in expression of genes for KVJ20 under different cultivation conditions and respective controls. Every value >1.5 and < 0.6 are in bold, and those with a significant increase is highlighted in dark grey. P = PKS, N = NRPS, NP = NRPS/PKS. *hgl*- heterocyst glycolipid, *bsl*- Suomilide-Banyaside -like, apt- anabaenopeptin, ncp- nostocyclopeptide, Sid?- putative siderophore, mvd – microviridin, Lan- peptides with predicted lantionine modification. Fold changes ≥ 2.000 are given in bold numbers.

	-N vs.+N		***Solid vs. Liquid***		***-P vs.+P***		***-Fe vs.+Fe***		***Competition vs. control***	
Gene cluster	***Liquid***	***Solid***	***-N***	***+N***	***-N***	***+N***	***-N***	***+N***	***20 vs.2***	***20 vs.10***
20-NifH	**2,697**	1,122	1,540	**3,702**	0,611	**2,487**	1,488	**2,871**	**2,202**	1,466
20-AvaK	**2,345**	1,004	**0,050**	**0,118**	**0,373**	0,659	1,745	**4,852**	**2,045**	**2,466**
20-GvpC	**0,469**	1,022	**0,076**	**0,035**	**0,039**	**0,063**	1,059	**0,274**	**3,968**	1,791
20-P1 (hgl)	1,368	1,254	**2,689**	**2,934**	**0,358**	**0,491**	**2,300**	**6,480**	1,616	1,356
20-P2	0,593	1,060	1,080	0,604	**0,166**	**0,124**	**0,157**	**0,090**	0,972	**0,440**
20-N1 (bsl)	**2,330**	1,073	**2,211**	**4,803**	**0,090**	**0,275**	1,536	**1,988**	0,775	1,130
20-N2 (apt)	1,084	1,110	**2,385**	**2,329**	**0,333**	1,597	1,788	0,844	1,137	1,142
20-N3	**2,093**	1,083	1,079	**2,087**	**0,357**	1,215	**0,508**	**4,356**	**2,367**	1,367
20-N4 (ncp)	**0,192**	1,214	**4,167**	0,660	**0,199**	**6,040**	**19,190**	**3,547**	**6,242**	**7,787**

20-NP1	**0,495**	1,069	0,566	**0,262**	**0,399**	1,124	0,753	1,846	1,818	1,572
20-NP2 (Sid?)	0,606	1,053	**19,977**	**11,498**	0,776	1,749	**2,408**	0,561	**0,107**	**0,321**
20- RiPP1	0,930	1,189	1,584	1,239	0,921	0,957	1,409	**2,684**	0,611	**0,428**
20-RIPP2	**2,276**	1,040	1,549	**3,392**	**0,443**	0,917	**0,142**	1,774	**0,506**	**0,386**
20-RIPP3	**0,204**	1,018	0,855	**0,171**	1,090	**0,249**	1,428	**0,254**	**1,945**	1,061
20-RIPP4	0,904	1,009	**0,494**	**0,443**	**0,179**	**0,178**	**0,165**	**0,126**	1,138	0,551
20-RIPP5	**0,287**	1,021	1,008	**0,283**	1,008	0,970	0,629	**0,340**	**3,318**	**2,622**
20-RIPP6	**1,788**	1,001	1,442	**2,577**	**0,173**	**0,374**	**0,068**	**0,424**	**0,456**	**0,484**
20-RIPP7	**0,488**	1,010	1,112	**0,538**	1,376	**2,938**	**0,497**	**0,591**	1,488	0,915
20-RIPP8 (mvd1)	1,128	**4,063**	**0,533**	**0,148**	**0,228**	**4,325**	**0,455**	**0,307**	**4,072**	**2,669**
20-RIPP9 (mvd2)	1,649	1,003	**2,398**	**3,943**	**0,123**	**0,489**	0,921	1,466	0,793	0,665
20-RIPP10 (Lan)	**2,469**	1,088	1,366	**3,100**	**0,082**	**0,181**	1,185	**2,575**	0,652	0,666
20-RIPP11 (Lan)	**0,331**	1,054	1,182	**0,371**	**12,964**	**12,607**	1,044	**0,367**	**4,811**	1,856

With regard to the expression levels in genes representative of their respective BGCs, we noted the following: Banyaside-suomilide -like gene cluster, 20-N1(*bsl*), was expressed at higher rates in cultures showing signs of nitrogen limitation, i.e. liquid $BG11_0$, on solid media, and with nitrate but without phosphate. Whether the suomilides (**1-4**) produced are somehow related to diazotrophic growth remains to be elucidated. A similar expression profile was noted for 20-RiPP10.

The anabaenopetin gene cluster (20-N2) was expressed at higher levels in colonies on solid media, which is line with observation made on high density cultivation of *Nostoc* [12]. A similar expression pattern was observed for 20-NP2 and the microviridin gene cluster 20-RiPP9.

NRPS involved in biosynthesis of suggested hormogonia inhibitor nostocyclopetide [13], 20-N4, was up-regulated under several different treatments, such as iron limitation, presence of competitor strains, in phosphate starved culture in BG11, and nitrogen deplete agar grown colonies. However, it has been shown that the transcription levels of *ncp* genes were not correlated to the release of the compound [13].

Several RiPP BGCs, 20-RiPP5, 20-RiPP8 (microviridin) and 20-RiPP11, were clearly up-regulated under co-cultivation with both competitor strains. In addition, the highest level of expression for 20-

RiPP 11 was observed under phosphate limitation. This may suggest that the products of these gene clusters are involved in extracellular interactions.

In summary, we can say that BGCs in *Nostoc* sp. KVJ20 are highly responsive to various environmental stimuli and show diverse expression patterns and is of high interest with regard to understanding the role of secondary metabolites in cellular differentiations, stress responses and interactions with other microorganisms.

Literature:

(1) Fujii, K.; Sivonen, K.; Adachi, K.; Noguchi, K.; Shimizu, Y.; Sano, H.; Hirayama, K.; Suzuki, M.; Harada, K.-i. *Tetrahedron Letters* **1997**, *38*, 5529-5532. DOI: https://doi.org/10.1016/S0040-4039(97)01193-3

(2) Ishida, K.; Christiansen, G.; Yoshida, W. Y.; Kurmayer, R.; Welker, M.; Valls, N.; Bonjoch, J.; Hertweck, C.; Börner, T.; Hemscheidt, T.; et al. *Chem Biol* **2007**, *14*, 565-576. DOI: 10.1016/j.chembiol.2007.04.006

(3) Ishida, K.; Welker, M.; Christiansen, G.; Cadel-Six, S.; Bouchier, C.; Dittmann, E.; Hertweck, C.; Tandeau de Marsac, N. *Appl Environ Microbiol* **2009**, *75*, 2017-26. DOI: 10.1128/aem.02258-08

(4) Mihali, T. K.; Kellmann, R.; Neilan, B. A. *BMC Biochem* **2009**, *10*, 8. DOI: 10.1186/1471-2091-10-8

(5) Mahlstedt, S.; Fielding, E. N.; Moore, B. S.; Walsh, C. T. *Biochemistry* **2010**, *49*, 9021-3. DOI: 10.1021/bi101457h

(6) Stanier, R. Y.; Kunisawa, R.; Mandel, M.; Cohen-Bazire, G. *Purification and properties of unicellular blue-green algae (order Chroococcales)* **1971**, *June*, 171-205

(7) Liaimer, A.; Jensen, J. B.; Dittmann, E. *Frontiers in Microbiology* **2016**, *7*. DOI: 10.3389/fmicb.2016.01693

(8) Pinto, F. L.; Thapper, A.; Sontheim, W.; Lindblad, P. *BMC Molecular Biology* **2009**, *10*, 79. DOI: 10.1186/1471-2199-10-79

(9) Argueta, C.; Yuksek, K.; Summers, M. *J Microbiol Methods* **2004**, *59*, 181-8. DOI: 10.1016/j.mimet.2004.06.009

(10) López-Gomollón, S.; Hernández, J. A.; Pellicer, S.; Angarica, V. E.; Peleato, M. L.; Fillat, M. F. *J Mol Biol* **2007**, *374*, 267-81. DOI: 10.1016/j.jmb.2007.09.010

(11) Potts, M.; Bowman, M. A. *Archives of Microbiology* **1985**, *141*, 51-56. DOI: 10.1007/BF00446739

(12) Guljamow, A.; Kreische, M.; Ishida, K.; Liaimer, A.; Altermark, B.; Bähr, L.; Hertweck, C.; Ehwald, R.; Dittmann, E. *Appl Environ Microbiol* **2017**, *83*. DOI: 10.1128/aem.01510-17

(13) Liaimer, A.; Helfrich, E. N. H.; Hinrichs, K.; Guljamow, A.; Ishida, K.; Hertweck, C.; Dittmann, E. *Proceedings of the National Academy of Sciences* **2015**, *112*, 1862. DOI: 10.1073/pnas.1419543112
